# Extra-Limites

**Published:** 1832-01-01

**Authors:** 


### EXTRA-LIMITES.

### DR. HACKET TO DR. JOHNSON.

*English Harbour, Antigua,*
30*th September*, 1831.

SIR,

As there appears in your Review for May, 1830, "Observations on the Blood," by Dr. Stevens, and as you decline any comment on that Gentleman's doctrines till you hear further from him, I am, in the mean time, induced to trouble you, and offer some remarks on the subject in question, which I am rather inclined to think will cause some surprise in your mind, when the unqualified assumption, set up by Dr. Stevens, for the successful treatment of fever in the garrison of Trinidad, by following (as he says) the practice inculcated by him in his Observations on the Cure of Malignant, Yellow, or Endemic Fever of the Tropics, is considered.

I regret, at the very onset, to appear uncourteous, in charging this Gentleman both with misrepresentation and want of candour, in what he has published; and, under these circumstances, cannot, I confess, suppress my surprise at his temerity, in inducing that very respectable and learned body, the COLLEGE of PHYSICIANS, to apparently sanction and give currency to his crude and visionary lucubrations, by the recital of his paper, officially it seems, amongst them. My object now is both to confute the misrepresentation of Dr. Stevens, and point out to you his very great want of candour; and, moreover, to prove that the sole merit, whatsoever it may have been, for the great success attending the treatment of fever in the garrison of Trinidad, belongs solely and exclusively to the military medical officers. I trust, ere I have done, to convince you that a borrowed garb has been assumed, which correct and proper feelings never should have allowed the Author of "Observations on the Blood" to have put on.

——— "Moveat cornicula risum
Furtivis nudata coloribus."

And if, from the nature of this affair, I may in any way appear an egotist, be pleased to set it down to the right cause, the ostensible situation I held, and not to any silly or unworthy vanity of my own. I have no wish to rob any man of the quantum of merit that is his due, but as I know of none due to Dr. Stevens which he so (let me say) unblushingly assumes for the treatment of fever in the garrison of Trinidad, it removes any delicacy on my part, in denying, in the most pointed and unqualified manner, his pointed assertions.

As to his Theory of the Blood, I wish to have nothing whatever to do with it here—considering it, independent of other reasons, one of those ephemeral "runnings riot" of the imagination, of late years greatly indulged in, by almost all the medical aspirants for fame; each wishing to locate or fasten fever, as it were, in some particular or peculiar organization of the human fabric, and each fancying he has caught it fast in that structure where it pleases him to presuppose that it rests or should rest.

As however I cannot admit the general correctness of the anatomical remarks on the blood contained in the publication of Dr. Stevens, and the whole data of his doctrine lying almost within the limits of his first page, I shall offer a few facts, drawn from dissection, that go to disprove the Doctor's Theory.

Permit me to inform you that every case that dies in our military hospitals must be opened and reported on; and therefore the opportunities of the military practitioner for examining the human body, and viewing the morbid changes in its structure, are perhaps greater than that of any private practitioner, however great or extensive his practice may be. On this account I may be entitled to some degree of credit for stating what I have actually seen, even though it materially differs from the statements made to the public. Be it remembered, my remarks are confined principally or altogether to Trinidad; the fever to be met with there, I may assume as the severer type of tropical fever; this place is even quoted by the Doctor, as being "generally considered one of the most sickly islands."

I shall now quote the passage referred to in support of the sanguineous fluid being the seat of fever.

"There is sometimes met with in the West Indies a malignant form of yellow fever in which, from the beginning to the end, it is evident from the symptoms that there is, during life, little or no affection of the solids, and often after death even the most able anatomists cannot detect any trace of disease, either in the brain, the stomach, the intestines, or any of those organs, whose derangement are generally supposed to be the cause of fever. In these fatal cases, there is no excitement in the commencement sufficient to injure the solids, and we can only ascertain the real cause of death when we open the heart, and examine the state of the once vital fluid; the cause of death then becomes evident, for we find there, in place of blood, a dissolved fluid, nearly as thin as water, almost as black as ink, and evidently so diseased, as to be totally incapable of either stimulating the heart or supporting life." "In both cavities of the heart the fluid is equally black, and in the whole vascular system, all distinction between arterial and venous blood is entirely lost."

In reply to this, my facts are these—that I never yet have seen a case of aggravated fever, (and I have seen, I may say, some thousands) in which, on dissection, one or other, most or all, of the following appearances were not present.

On removing the calvaria, the sinuses and blood-vessels appeared turgid with dark *viscid* blood—the substance of the brain itself was generally firm; often hard and turgid with blood; its medulla studded with innumerable red spots giving out its sanguineous fluid at each cut of the scalpel. There was often effusion in the ventricles; the plexus choroides generally injected, or, in fact, like a congeries of blood-vessels—effusion was also found, in basi cranii, descending along the medulla oblongata to the medulla spinalis. It was no uncommon occurrence to find coagulable lymph between the dura and pia mater. The lungs were either sound, or exhibiting marks of previous disease; but gorged with thick, fluid, dark-coloured blood. The heart was natural, or large and flabby, with effusion in the pericardium, whilst the auricles and ventricles exhibited a thick dark-coloured *grumous fluid.* The stomach was generally distended with flatus; sometimes presenting a seeming contraction across from its great to its lesser curvature—its villous coat disorganized, in patches easily rubbed off by the handle of the scalpel—either deeply tinged with a black matter, or containing in its cavity some ounces of a dark coffee-coloured fluid, totally dissimilar to the homogeneous yet grumous-like blood, the former being stringy and much more fluid in its nature, and not in any manner presenting the same materiel as exhibited by the latter fluid. The liver, in some instances, was preternaturally large or small; in colour, mottled grey, or a deep somewhat purple hue—the blood-vessels filled with a dark fluid—the cystic duct was found most frequently impervious, so that even a bristle could not find entrance; whilst the cyst was itself found greatly enlarged, filled with a tar-like matter in colour, and almost so in consistency—or perhaps it was occasionally found to contain a thin brownish watery fluid. The spleen was either preternaturally enlarged, crumbling under the fingers, or preternaturally small and hard. The coats of the smaller intestines partook more or less of the appearances of the stomach—whilst, what struck us most, was the very frequent contraction of the colon, which was most extraordinary throughout its whole course: not larger in calibre than the diameter of one's thumb. This, with the contraction of the cystic duct, most rivetted our attention; so that with the epigastric sensibility and careful examination of the abdominal parietes, we not unfrequently made a tolerable correct prognosis of what was occurring within. The pelvis of the kidney was sometimes involved. The ureters or bladder seldom, to my recollection, partook of disease.

The inference to be drawn from the foregoing is, according to my conception, that the solids and fluids are equally contaminated, under the effects of fever. Let it not be supposed that the contracted state of the colon, or impervious state of the cystic duct, were effects from long-continued disease alone—no such thing—for, I have seen these appearances indiscriminately, as well in those who have died in eight and forty hours from the attack, as in those who lived to the fifth or seventh day. It will be perceived (independent of the other great and striking differences) that the *watery thin black ink* blood spoken of so confidently by Dr. Stevens, was *never detected in our dissections;* but I nevertheless have seen, through the progress of the disease, the blood ooze from the gums, eyes, anus, &c. &c. as stated by him. It is also to be observed that the blood, after venesection in fever, seldom presented, in comparison to the frequency of the operation, a cupped or buffy coat. The coagulum had not tenacity, and was easily broken down by the finger: but, I repeat, on examination after death, the appearances the blood presented, were invariably, and without one single solitary exception, dark coloured, *viscid* or *grumous*, as stated by me; though, in saying this, I would not be understood as denying the truth of the assertion altogether, as an occurrence never, under any circumstances, happening; for in the Principal Medical Officer's Office at Trinidad, the Dissection Reports of the York Rangers stationed there in 1816, gives the effects of what Dr. Jackson calls the *cachectic form of fever*, peculiar to that island. These cases all died *œdematous*, after a long protracted term of suffering, with pallid leucophlegmatic countenances, and with hearts, in several instances, enormously enlarged, and dark watery fluid blood in *the veins*. I mean to say, that what is stated even by Jackson, is not a common, or by any means a common, or even a very frequent occurrence, in tropical fever; and I am thus bound to impeach Dr. Stevens's candour, for stating in support of his hypothesis, what he has done—viz.—a marked—a peculiar characteristic—never as it would seem absent—always present—thus giving a worked up fancied picture, calculated only to mislead those who can have no opportunities themselves of witnessing fever in this climate.

I appeal to all my naval and military brethren—I appeal to yourself, who have ably written on the diseases of this climate, as well as to all the civil practitioners who have practised here, for the truth and correctness of my statement. I appeal to each and all, if they do not recognise in my description what they have more or less invariably noted themselves.

At No. 2, page 5, the learned gentleman seems to me to attempt to prove too much for his own credibility, by asserting that "the colour of the whole mass of blood, both in the *arte ies* and veins, was changed from its natural scarlet or modena red, to a dark black."

In the *living artery*, I unhesitatingly deny this assertion; for, when, even in the latter stage of endemic fever, for some affection of the head, arteriotomy of the temples was resorted to, the blood invariably presented the bright scarlet hue, its peculiar characteristic. As to the blood in the dead arteries, this is the first time I ever heard it asserted that blood was contained in those vessels after death, in tropical fever. I am supported in the negative I now give to such being the case, by the opinion of numerous medical practitioners of different West India Islands with whom I have conversed on the subject.

Dr. Stevens's hypothesis seems to be little different from what Dr. Paris states in the historical introduction to his "Pharmacologia," under the head "CHEMISTS," and "CLASSIFICATION of REMEDIES." Dr. P. theie pointedly shews, "the fatal errors into which such an hypothesis was liable to betray the practitioner," illustrated in the history of the memorable fever that raged at Leyden in the year 1699, and which consigned "two thirds of the population of that city to an untimely grave, an event which, in a great measure, depended upon Professor Sylvius de la Boe, who, having just embraced the chemical doctrines of Van Helmont, assigned the origin of the distemper to a prevailing acid! and declared that its cure could alone be effected by the copious administration of absorbent and testaceous medicines."

I shall not (as I have already stated) dwell here on Dr. Stevens' doctrines; but leave his refutation in the recorded opinions of the able writer of the Pharmacologia.

However common it is at the present day for every man to have a "flight of fancy" about fever or its seat, I nevertheless cannot forego giving an epitome or sketch of mine, however humble it may appear, acknowledging at the same time, that I profess to know but little of that disease, further than the phenomena it exhibits, and the mode which experience has taught me of encountering it. This also teaches me that the temperament or idiosyncracy of the individual attacked often greatly modifies both symptoms and degree, or perhaps I might even say occasionally the type of fever. For instance, in one will be found intermittent or pure remittent; in another, a very irregular intermittent; and in the next, perhaps, may be found typhoid characteristics mixed up with the remittent type. This also accounts to me for the anguish complained of in different parts of the solids, by different patients—thus the head and stomach (accompanied with great torpor of the intestinal tube) are generally the parts most affected in tropical fever, with distressing pains in the back, and calves of the leg. Yet these I have seen diversified in different patients—whilst, in some of the most fatal cases I have seen in the tropics, the stomach, from the onset, seemed the sole and only organ distressed—nay the only one complained of, the head being not affected at all—the integrity of the sensorial powers being preserved to the last.

Viewing fever, therefore, for my own part, as most generally, or rather always characterised by a suspension, or even cessation of all or part of the healthful or natural secretions, I am forcibly impelled to the contemplation of Philip's and Brodie's experiments on digestion and circulation; and to infer that the nervous influence (of the *par vagum, at the least*) is impeded ; hence the human frame suffers more or less, in all or most of its component parts. I am convinced that to restore any of these secretions, no matter which, even let it be but the secretion from the pituitary membrane of the nose, leads to the most certain results. Let secretion be accomplished, and abatement of fever, I *know*, will invariably follow ; whether its supposed seat be in the mucous, nervous, or any other favoured tissue, or in the blood itself. This is my brief and simple commentary on fever, and this view of it has led to most efficient and successful practice. But in our practice, we must be prompt and decisive ; aiming to cut short the attack even in the first 24 or 48 hours, (for it is only within this period we can really count on our art)—this period once allowed to pass, without an impression being made, the disease becomes as it were a labyrinth, most difficult to unravel. The remedial means which experience has taught us to be most effective, are those which act on the secreting organs, for 'tis through these organs that the balance of the circulation is lost.

Experience—I might say sad experience—has proved this to me—the writings of the excellent and observant Jackson taught it to me, for his has been principally my text book : from him on this subject (with becoming gratitude I say it) I have learned much, and next to him perhaps I owe much to Mr. Tegart, for having, by his circular addressed to the medical officers in the command, when Inspector General here, drawn my attention to the great advantages to be derived from the administration of croton oil in tropical fever. I now candidly acknowledge the *great* and *satisfactory advantages* we derived from *his* suggestions and observations, and further that I availed myself of them *to a great extent*, and I *faithfully* and *conscientiously believe to the great preservation of human life*. I also feel indebted to a junior member of the department, Staff-Assistant Surgeon Cowen, for directing my attention to the administration of large doses of quinine in the intermission or remission of fever, which was attended with singularly happy results.

As Principal Medical Officer of Trinidad, for nearly five years, I know of no one to whom I am indebted for any professional hint, or to whom that garrison owes any thing for the great preservation of human life under pressure of disease.

I have now to state that Dr. Stevens visited Trinidad some time in 1828—when he also visited St. James's hospital there—our practice was then explained to him by Mr. Greatrex—for I did not see him,—this it seems elicited from that gentleman the remark " of the similarity of our practice to his own"—the only difference he observed between us (and I think Mr. Greatrex added, he remarked it was of little consequence, for we were in the habit already of using alkalies dissolved in toast-water for common beverage, as directed by Jackson) was the preparation of potasse *principally* used—we using the supertartrate (for we were in the habit of giving this remedy to a very large extent —half, and ounce doses) he the carbonate or subcarbonate of it, or soda, I forget which, remarking too, on the great advantages of using the lancet *in the* warm bath, and seeming, from what I understood, to make *that* the sheet anchor—and saying, since he adopted it (bleeding in the bath) he had lost no case of fever, whereas before, he lost many. This is laid down by Jackson, almost as a *sine qua non*, and was the routine of our hospital practice.

Mr. Greatrex also told me then of a theory of Dr. Stevens', " of an acid in the blood;" and mixing some blood and soda in a porringer, was inclined to think the appearance therefrom seemed to corroborate the idea. I laughed, and said " do you suppose that blood in the porringer possesses the same properties which it did, when within its proper vessels? Assuredly not—therefore how incorrect any inferences must be, drawn from such premises; for, whatever affinity these substances may have for each other, when thrown and thus mixed together in a cup; yet, I am stupid enough not to comprehend how the same thing can take place in the living fluid, enclosed in its proper tubes. Considering, too, the very minute quantum of soda that can possibly pass from the stomach *direct* and *unchanged* into the *mass* of *circulation*, how small must be the means to produce such mighty effects." I do not believe that the subject thus treated was ever again renewed between us.

" In August, 1828," Dr. Stevens states, "at a time when there was a good deal of sickness in the garrison of Trinidad, this practice (giving saline medicines) was adopted in the military hospitals of that island;" (mark!)—"that is to say, they bled freely, and used active purgatives in the commencement, to reduce the excitement, and afterwards the saline medicines were administered, until the fever abated, and during the convalescence the quinine was given in large doses." This was really our practice *both before and after the Doctor's visit;* but there appears to me an insinuation in the above quotation, or even probably more than an insinuation, implied in it, viz. that we were indebted to him for this mode of practice. This is not directly said, but I appeal to you if 'tis not left to be understood; yet Dr. Stevens had not the candour to give *the whole of Mr. Greatrex's letter,* which bears principally on the activity of the above practice, as inculcated by Jackson, aided chiefly by croton oil and quinine, and to which activity his letter attributes our success;—nor, by the bye, has he the common candour to state *what* he found our practice to have been; but gives a *garbled* extract, to suit his own purpose, from Mr. Greatrex's communication, not even stating, (which was remarked by Mr. Greatrex to him and worthy to be noted,) "that in those who died, though *dosed with soda,* the blood presented no difference as to hue or consistency, from that found on dissection in others."

'Tis painful to me thus to express myself; if I do an injustice, by my interpretation of Dr. Stevens's statement, I am truly sorry for it; and would call on him to correct it or set me right; but, I confess, in the light I now view it, never was daw bedecked in falser plumes than Dr. Stevens has arrayed himself; and, as proof of the accusation which I have made throughout this communication, both of misrepresentation and want of candour, I herewith send you a copy of Mr. Greatrex's letter to Dr. Stevens, on which that gentleman would seem to found the principal data of his hypothesis, and from which it appears that there is not one iota in it, that can fairly be said to be at all corroborative of his statement. So much for candour and professional integrity!

In pursuing this subject, 'tis but fair to say that a combination of mur. et carb. of soda, with nitre, was frequently and commonly given, as well as alkalized toast-water and supertartrate of potass—the former was the wish of Mr. Greatrex, and the recommendation of Dr. Stevens, to which I had no objection; as I considered it tended to the fulfilment I sought—namely, renal, cuticular, or visceral secretion. These secondary means were, however, scarcely ever administered within the first 24 or 48 hours of almost any of the heavier or more dangerous descriptions of fever—latterly, certainly not, being merely considered adjuncts, and prescribed when febrile excitement had nearly or altogether subsided; but when some thirst or lurking febrile heat was still perceptible to the touch. Under these circumstances, the medicines were given to keep up the already aroused secretions. But, as to considering them specifics, or possessing annihilating febrific powers, or extraordinary virtues *per se*—the power, for instance, to unlock the cystic duct, most frequently impervious, or to relax to its natural dimensions the contracted colon, or even to change the consistence or colour of the blood, in those whom I shall term "*sodafied,*" was about as ridiculous as the experiment of mixing up blood and soda in a porringer. Culpable indeed, it strikes me, must that practitioner be, who, in West India fever, would place his reliance on such *trumpery*—on means so inadequate to the proposed end!

Although Mr. Greatrex gives, in his letter, a general view of our practice, as established at Saint James's Hospital, I shall, nevertheless, here exhibit a condensed sketch of it also;—and, as Dr. Stevens says, "they bled freely and used active purgatives," let us inquire to which of the remedial means we were indebted for success? In the following will be recognised Jacksonian practice; viz. an emetic of zinci sulph. on admission—bleeding *in the* hot-bath, most frequently ad deliquium, though not invariably—on coming out, friction and dry rubbing—after the operation of the emetic, compound jalap powder—soon afterwards enemata, composed of salts and castor oil, were thrown up to the extent of *three or four, or even I have known five large syringes full;* or, as much as the torpid intestines would hold, and as quickly in succession as they could be administered—all this was accomplished in a few hours. Towards evening, if there was no abatement of fever, the shower-bath—then followed bleeding again. As night came on, there was often great restlessness, with distressing pains of head, back, and calves of the legs, (very common symptoms, and indicating, I imagine, that the solids were somewhat implicated)—great irritability of stomach, or incessant vomiting. In such cases, croton oil was invariably given, to the extent of three or even four drops. I have known this repeated thrice through the night, and it is very worthy of remark, that, the more irritable the stomach, [though, *prima facie*, to those unacquainted with the great febrifuge virtues and extraordinary powers of croton oil, in restoring the proper peristaltic motion of the intestines, which seems, in this disease, to be altogether inverted, this irritability, the very leading feature and peculiarity of tropical fever, would be a cogent reason for not administering the oil,] the more invariably was the croton oil found to be beneficial—so much so, that, in the morning, we generally found our patient, thus treated, either enjoying a perfect, or nearly perfect remission. Twelve grains of sulphate of quinine were then given, in a little syrup, or in solution with sulphuric acid; or if, from appearances, we were not satisfied of the permanency of the remission, a drop, or perhaps two, of the oil of croton were given with the quinine in syrup; or four or five drops in an enema;—whilst the body was sponged with either cold or warm vinegar and water, as circumstances seemed to demand. After such active treatment, we might as well consider the application of a blister—the lavements of cold water, often used in our practice in the course of fever—the erect position in bed, with cold cloths to the shaven head—or cupping the nape of the neck or epigastrium—or any other of our subordinate measures:—as well, I say, might we suppose *any single one* of these various means to be *the sole* cause of the cure, as that a little soda produced all the advantages which we obtained—and this too from the wonderful discovery of Dr. Stevens!! There is something—I hardly know how to characterise it—throughout all this affair, which I should term—at all events consider—not very unlike a PROFESSIONAL PETTY LARCENY, and which—

> "By Heaven I'd rather coin my heart,
> And drop my blood for drachmas,"

than be guilty of!

In corroboration of what, throughout this paper, I have advanced, as to remedial means, I shall add, that I was directed by the Inspector General, Dr. Baxter, to proceed from Barbadoes to Tobago, in October last, in consequence of fever of a fatal character having broken out in the garrison there, and the following extract is from my official communication to him on the subject, dated Tobago, October 23d, 1830.

"As to the character of the fever, it appears to me to be the aggravated congestive form; and nothing different from what I have been accustomed to see for the last five years, at Trinidad. These cases do not bear, at certain periods, the *free* use of the lancet; and though it is requisite, yet, as laid down by Jackson, it requires particular caution in its application."

"I am glad to say that the practice followed at Trinidad has been, (if from so few a number a fair criterion can be established) successful here also. May I remind you that bleeding, according to circumstances, in the hot-bath; filling the torpid intestines with purgative lavements—croton oil to the extent of four drops; given also in enemata, and seizing the first remission to administer a ten grain dose of quinine, the shower bath, &c. in the progress of the disease, were the general characteristics of our practice."

If I have been betrayed to dwell longer on the foregoing subject than may perhaps be thought necessary, let it be remembered that it is difficult, on some subjects, to abstain from being diffuse or egotistical, particularly when "one's hard and honest earnings," are publicly claimed by the undeserving;—and, in passing judgment on what I have stated, let there be carried to account, the *never ceasing toil and anxiety* devolving on the medical officers, both day and night, in attendance on, and watching by, the bedside of the sick, in tropical fever, under a severe and heavy invasion of it in a garrison. Opposed to all these, let a verdict (if the profession think fit) for the successful issue of the treatment, be triumphantly recorded by a *mere casual visiter*, viz:—"SODA" *versus* ALL OTHER MOST PROMPT AND VIGOROUS MEANS, pursued by those in charge of the sick—and at the very couch too of suffering; nay, I may even say, themselves in the very "battle's front" of disease! Such circumstances impartially considered, will perhaps prove some little palliation, for whatever may, at first view, appear objectionable, either in the manner or matter of this communication.

I am Sir, with great respect,

W. HACKET, M. D., *Surgeon to the Forces*.

English Harbour, Antigua,
*30th September*, 1831.

To Dr. JAMES JOHNSON.

("Extract") Mr. Greatrex's Letter alluded to in the foregoing.

Copy. SAINT JAMES'S, TRINIDAD, 12*th May*, 1829.

Sir,

"I have reflected on the statement you made to me of your theory of West India fever—made experiments with the alkalies—and administered the mixture, composed of muriate of soda, nitrate of potass, and water, in the proportions and at the times you pointed out; but with what particular effect, I regret to say, I cannot determine for you, in consequence of its being mixed with the effects of several other measures which we are in the habit of adopting. I have been desirous to make out the precise uncombined consequences on the living blood of your soda mixture; but I will give you facts. During the month following your visit to us, we admitted to hospital 40 or 50 cases of the remittent or climate fever, of which three died; and, I administered the soda mixture myself to *a given number* in order to be sure that it had been taken. It was of *that number* that the three died. One of them was admitted within the first 18 hours, another on the second day, and the third on the third day. After death, the blood was in a state of *solution*. Examination was performed 12 hours after decease; the blood had not been alkalized by the soda; it was dark-coloured. The morbid evidences were *precisely similar to those showed you in the Dissection Reports.* These men had been in the habit of drinking immoderately of rum, and undergoing unusual fatigue; exposed directly to the solar heat. By the action of the alkalies on the blood *abstracted* from cases of remittent fever, it is plain enough there is much acid in it. On that of patients of the climate fever the same observation occurs, and on that of the *healthy man* here, (at least so to all appearances,) a similar effect results; but less in degree from the alkalies. I have not, as yet, tried the alkalies on the blood of animals in this locality so productive of malaria, which I presume is to be considered the cause of the altered condition of the blood. The remittent fever blood is darker coloured and thinner than the blood of fever-cases in England. As you remarked, it seems too liquid, as if dissolved. It never presents a firm crassamentum, nor any of the buffed or cupped appearances, so commonly exhibited by fever blood in England. The same circumstances obtain in the blood of the climate fever, (as you very appropriately designated the yellow fever,) also in the colour and consistence of the uncomplaining man's blood, generally speaking; but sometimes more florid; yet still being thinner than what healthy blood is in England. I have taken the latter as the healthy standard. *A similar change from purple to scarlet, as by the admixture of the alkalies, is occasioned in the blood, by immersion of the patient in the hot-bath, as seen by bleeding during, or immediately after, his removal from the bath.* Do you think that this might be explained by saying the acid and aqueous parts of the blood have been driven off as perspiration by the hot-bath? I purpose trying the effects of the alkalies when I return to England, which may perhaps assist in coming to a conclusion.

The way we combat fever, is agreeably to Dr. Jackson's system, so admirably set forth in his invaluable book, viz.—We place the patient in a hot-bath at 110 degrees, and when well heated, and he perspires, open a vein, and take away from 36 to 60 ounces of blood—lift the patient on the bed, and have him well rubbed all over for 10 or 15 minutes—the head is placed tolerably high; in most cases a sound sleep supervenes, which continues from one to five or six hours, during which the cutaneous exhalations continue. On waking, fresh linen is put upon him and on the bed; an enema of sulphate of soda and castor oil with water is now administered, to the extent of two gallons, more or less according to the degree of peristaltic action of the intestines which may be induced. After the evacuation of scybalous and blackish compounds, your soda mixture is given every fourth hour. From the time of his waking, *now*, or in a few hours, we administer the croton oil, agreeably to the directions published by Mr. Tegart, in the London Medical and Physical Journal for August, 1825; and the effects of this oil, as they have appeared in some hundreds of cases which have come under my observation in Trinidad, fully support the high merits which Inspector Tegart has assigned to it. The night is passed sometimes with, sometimes without sleep; the air in the wards of the hospital is never suffered to become close or offensive. Your mixture is continued through the night—the next morning, at 6 o'clock, in 99 cases out of a hundred, even including what is termed climate-fever, there is a remission of symptoms, more or less decided, when we give from 12 to 18 grains of sulphate of quinine in one dose, instead of three or four grains every fourth hour. Never any unpleasant effects in the sensorium (as many authors have said) have followed the administration of the sulphate of quinine, in our hospital. The alkalized toast-water, ad libitum, *which Jackson advises*, is used as a common drink, as is also the supertartrate of potass dissolved in water. As the temperature of the day rises, the symptoms seem inclined to recur, when we put the patient under a shower-bath, two or three shocks—then place him in a current of air, and apply cold-wash to his head. The effect is decisive. On the third morning of his being in hospital there is almost an intermission of symptoms, when we merely repeat the dose of the sulphate of quinine. If any vomiting recur, we give another dose of croton oil, and if any local pain or headache remain, we apply from eight to ten cupping glasses, and abstract from 20 to 30 ounces of blood—then, if necessary, apply a blister, but the cupping is generally sufficient. We were led to give the large dose of the sulphate quinine by a medical officer touching here, and telling us that it was given in that quantity on the Coast of Africa, with the best effects, and such have been the effects here; two doses are all that are necessary. On the fourth day of the patient's being in hospital, convalescence is established, when we give an ounce or two of porter frequently, in conjunction with light soup, an occasional shower bath—and keep the alimentary canal free. Under Divine permission, the above system has been applied to 340 cases, or thereabouts, including both the remittent and yellow fevers, admitted to hospital, after the fever had existed variously from 6 to 72 hours, antecedently to an application at the hospital, with such success, that, during the last seven months, not a case has died. Men have died in that period, having the remittent fever; but, at the same time, having abscesses in the lungs, and purulent expectorations.

I have the honor to be, dear Sir,

Your most obedient humble servant.

(Signed) E. GREATREX.

WILLIAM STEVENS, Esq. Surgeon, St. Thomas's.

The following extract of a letter from Mr. Greatrex (after perusing a communication of mine on the foregoing subject, and essentially the same) I beg to append—'tis necessary, in explanation of certain passages, to state, that Dr. Stevens wrote to a military friend belonging to the garrison of Trinidad, though *not a professional man*, to procure from Mr. Greatrex answers to certain queries which he put to him.

("Extract.") NAVAN BARRACKS, Ireland, 24*th July*, 1831.

"I have carefully read Dr. Hacket's letter, and have to observe that it is correct in all its parts; and that I concur in the view he has taken of Dr. Stevens's conduct.

I enclose, for your perusal, my letter and note to Dr. Stevens, as taken from a copy of the originals, and forwarded to me at Barbados, early last year, by my request, in consequence of the queries from Dr. Stevens alluded to in Dr. Hacket's letter.

I withheld, as you have seen by Dr. Hacket's letter, all authority for the publication of *a part* of my letter—*still Dr. Stevens published a garbled extract from it.*

I have not the queries, but if possessed, they would be unnecessary, as you have perceived their purport from my remarks upon them in reply to Dr. Hacket.

The first and second queries were clearly meant to produce, that which might have appeared to those who are unacquainted with the healthy and sickly periods in the West Indies, the belief (intended to be produced by Dr. Stevens) that we must have adopted *his system of practice* in Saint James's hospital; because the corps became healthy shortly after his visit. I have often thought (at least it is charitable to think so) that it was probably this want of observance of the effects of the seasons upon the British soldier, which caused the correspondent of Dr. Stevens to fall into the mistake, in writing what *he was not in any degree warranted in doing, by the state of the case.*"

(Signed) "E. GREATREX,"

Assistant Surgeon, 12th Lancers.

### II.
### CHOLERA MORBUS.

To Sir HENRY HALFORD, Bart. M.D., President of the Board of Health, &c.

Sir HENRY,

When the disease misnamed Cholera Morbus was confined within our East Indian dominions, it concerned us only as a remote evil—but now that it has traversed Asia, invaded Europe, and advanced to the shores of the Northern Ocean, we awake from our dream of security.

"Tua res agitur proximus cum paries ardet."

The Government has adopted measures suited to the emergency. A Board of Health has been instituted, and Medical Commissioners have been sent to Russia. In August last, the Board published "Papers on Cholera," among which are letters from Drs. Russell and Barry, dated St. Petersburgh, July 27, 1831; in which it is unquestionably shown that the Russian plague does not differ materially from the Indian; at the same time it must be admitted that their identity is not satisfactorily made out. They have diagnostic marks as specific, at least, as those of Variola and Varicella, vulgarly recognised by the appellations of Small Pox and Chicken Pox, which diseases, though they have molested the inhabitants of this world for centuries, are still confounded by the Members of Boards and Professors of Pathology.

There is one thing clearly proved, that both assume the most alarming aspect at a certain period, when, if the most vigorous and skilful exertions be not made, both will speedily terminate in death. It is to be regretted that we cannot reconcile the fatality with our notions of skill; nor have we as yet any reason to entertain high expectations from the labours of the Commissioners, even subjected to the sagacity of the Board. We must have something more intelligible to guide us than the directions lately promulgated, otherwise, in the event of a visitation, we should have less to apprehend from the disease than from the treatment.

To suppose that this disease will visit the British islands is not unreasonable; the present epidemic reminds us of one which took a course not dissimilar in the reign of Edward III., anno 1349.

"A destructive pestilence," says Hume, "invaded England, as well as the rest of Europe; and is computed to have swept away near a third of the inhabitants in every country which it attacked. It was probably more fatal in great cities than in the country; and above fifty thousand souls are said to have perished by it in London alone. This malady first discovered itself in the north of Asia, was spread over all that country, made its progress from one end of Europe to the other, and sensibly depopulated every state through which it passed."

Extraordinary distempers also occur, seemingly confined to the country in which they originate, of which we have a signal example in the reign of Henry VII., anno 1485.

"There raged," says the same historian, "at that time in London, and other parts of the kingdom, a species of malady unknown to any other age or nation, the sweating sickness, which occasioned the sudden death of great multitudes, though it seemed not to be propagated by any contagious infection, but arose from the general disposition of the air and of the human body. In less than twenty-four hours the patient commonly died or recovered; but when the pestilence had exerted its fury for a few weeks, it was observed, either from alterations in the air, or from a more proper regimen which had been discovered, to be considerably abated."

The latter malady is ascribed to the state of the air, and the disposition of the body conjointly, and to the former the same causes may be with equal cogency transferred. Whether, therefore, the cases said to have recently appeared in Sunderland, Newcastle, and London, are, or are not, of the Asiatic description, unless you chain the air, or command the winds, you can oppose no obstacle to the march of the dreaded enemy; if the ensuing Spring should be moist and chill, with south-east winds predominating, a visitation may be apprehended, in contempt of all guards, quarantines, and *sanitary cordons*, the relics of barbarism; but if a melioration could be effected in the constitution of the atmosphere, this would be at once a universal preventive and remedy. Taking a hint from antiquity, might we not have fires on our hills, and discharges of artillery throughout the land?

With regard to any specific cause of the Cholera, I think every problem relative to it most easily solved, upon the supposition of a noxious temperament of the atmosphere; with regard to its contagiousness, I refer to the able letters of Dr. James Johnson of London. I may just observe, *en passant*, that in this instance the doctrine of contagion, like the despotism of Turkey, seems to me to have for its supporters terror, prejudice, and ignorance; let us proceed to matters of immediate interest, let us devote our minds to attain some fixed principle to regulate our proceedings, should this calamity, which heaven avert, render it necessary for us to exercise the duties of our profession.

Independently of the general advancement of pathology within the last twenty years, any one who can appreciate the knowledge acquired of the Indian Cholera itself, within a much shorter time, must be astonished at the apparently inert inductive faculties, and the consequent indecision of well informed and intelligent men, such as those are who compose the Board of Health.

It may require explanation, why I, who never was in India, should hazard an opinion respecting Indian Cholera; on this point you will, I trust, find the following brief yet circumstantial narrative quite explicit and satisfactory. Above thirty years ago, I commenced a series of anatomical investigations concerning the changes which proceed in the centre of the nervous system during diseases, constitutional, general, and local. I am not aware that such an undertaking had ever been previously devised. Though my opportunities were few, I omitted none, and by the year 1808 I had collected a mass of facts illustrating affections distinguished by the epithet nervous, which I then communicated in my lectures; by the year 1814, I had examined the brain and spinal marrow, and their nerves individually, from the olfactory to the last of the cauda equina, in relation to diseases of every class, including fevers, contagious and non-contagious, inflammatory and putrid, eruptive and non-eruptive; partial statements of the results crept into periodical publications, and inaugural dissertations from the year 1810 to 1820. In this interval, some medical men of eminence began to see that a new avenue had been opened to the arcana of the animal economy; since then, in every quarter of the globe, the spinal marrow has been attended to, and of late, what I many years taught to a few has obtained universal assent, viz. "that unless the brain, spinal marrow, and their nerves, be examined conjointly, and in connexion with the other organs of the human structure, all anatomical results will not only be incomplete, but must fail to throw any other than a delusive light upon the most common, numerous, and dangerous diseases."

In 1819, Dr. Robert Wight, an ingenious and ardent cultivator of medical science, went to India, and verified my suspicion, that Cholera was chiefly a spinal affection. He was, what probably no other man then in India was, expert in anatomising the spine, and well acquainted with the changes which the spinal chord exhibits; were metaphorical language admissible, I would say that he burst the barriers, and exposed the strong hold of the Asiatic destroyer.

The first essays of Dr. Wight concerning Cholera are to be found in a folio volume printed at the Asylum Press, Madras, 1824, consisting of select cases and observations, and entitled, "Report of the Epidemic Cholera, as it has appeared in the territories subject to the Presidency of Fort St. George, drawn up by order of the Government, under the superintendence of the Medical Board, by William Scot, Surgeon and Secretary to the Board." The Doctor concludes this communication to the Medical Board as follows:—"These conclusions are not deduced from *a priori* reasoning, but from facts first ascertained and brought to light by the scalpel. They were first drawn by Dr. Sanders of Edinburgh, with the morbid parts before him; they are of great practical importance, as they direct us to the very point on which to apply our remedies in these diseases; they show us where local depletions can be most advantageously made in those states of the system where extensive general ones would certainly be fatal. They show us also how we can most effectually assist the operation of general with local remedies, by indicating the 'locality' of the disease; and lastly, they show the states of the system, in which large general depletions can be used with safety and advantage, and where they are inadmissible."

The method of examining the contents of the cranium and spine, in connexion with those of the larger cavities, which, as already related, I was the first to follow in Europe, and Dr. Wight in Asia, is now, we learn, adopted in Russia. In the

"Papers on Cholera," we have the report of Dr. Keir on the Cholera of Moscow, which prevailed there in the Autumn of 1830 and Winter of 1831, in which he gives a very lucid and accurate detail of the *post mortem* appearances. We have also a document, the production of Drs. Russell and Barry, in which they detail the symptoms, from the beginning of what they call the first cold or collapse stage, to the end of what they call the second stage, that of re-action, heat, and fever; but their communication might have been of infinitely greater utility, had they inquired into the condition and feelings of the patients before the accession of the cold stage.

So graphic is the description given by Drs. Russell and Barry of the cold or collapse stage, that any man who had the requisite anatomical knowledge, on reading it, would instantly detect general paralysis blended with spasms, and ascribe it to the spinal chord, which was involved from its connexion or roots within the skull to its termination in the loins; and observing that, from the mouth to the toes, all the functions of motion, voluntary and involuntary, including those of secretion, were more or less suspended, arrested, and disturbed; that, in fine, that centre whence life emanates suffered almost as minutely as when labouring under severe concussion; he would determine without any great effort of reflection, that the treatment of each must be conducted on the same principles. It occurred, however, neither to the Russian Physicians, British Commissioners, nor to our Board of Health, that the very first thing to be attempted in the stage of collapse, was to relieve the centre of the nervous system, and thus restore the free communication of its influence to all the parts of the organization; which proves that, in their reasoning, they were not accustomed to combine the symptoms of disease, with *post-mortem* appearances. We may remark, that were the accurate observation of symptoms the only thing necessary to the knowledge of disease, the ancient empirics would have perfected nosology. But those, whom we have mentioned above, had an advantage which the ancients had not—they had the anatomical facts to illustrate the symptoms—they had two sides of the triangle, and could not find the third—they did not perceive the nature of the disease, and consequently could not conceive or divine what ought to be its remedies.

We shall next proceed to the Russian Cholera. In the report of Drs. Russell and Barry, there are assigned to it certain occasional preliminary symptoms, and others "which strongly mark the disease in its first cold or collapse stage, and a third set which characterise the second stage, that of re-action, heat, and fever." Of the preliminary symptoms, "Diarrhœa, at first feculent with slight cramps in the legs, nausea, pain or heat about the pit of the stomach, malaise, give the longest warning. Indeed, purging or ordinary diarrhœa, has been frequently known to continue for one, two, or more days, unaccompanied by any other remarkable symptom, until the patient is suddenly struck *blue*, and nearly lifeless. Often the symptoms just mentioned are arrested by timely judicious treatment." With the accession of the blueness, their first stage is completely formed, in which "the lips, the face, the neck, the hands, the feet, and soon the thighs, arms, and whole surface, assume a leaden, blue, purple, black, or deep brown tint, according to the complexion of the individual, varying in shade with the intensity of the attack." Further, they inform us that "the singular malady is only cognizable, with certainty, during its blue or cold period," and that, after re-action has been established, *it cannot be distinguished from an ordinary continued fever*, except by the shortness and fatality of its course."

The preliminary symptoms here given are common in fevers, merely constituting a part of the cold stage; and in having cold and hot stages, this disease differs not from fevers in general. Even in "the intensity" of their (Drs. Russell and Barry's) first stage there is nothing peculiar. That man is, indeed, but little conversant in the history of medicine, and has accumulated no great store of mental wealth from practice, who has not read of, and seen the same things on many occasions, and from various accidents, as well as in malignant fevers; and who did not observe, that when the central oppression was removed, the discoloration vanished, the re-action was mitigated or averted, and the patient saved. Nor is there any thing peculiar in the appearances discovered by dissection, since, in all fevers, except the hectic and symptomatic, the spinal marrow is similarly affected and deeply involved. To sum up, *the Indian or Russian Cholera is no other than an epidemic fever;* which, we may add, is very ill named, both because it is a fever, and because the term Cholera signifies a superabundance of bile, though in this disease the bile is either deficient or wanting.

We now come to the point of vital importance, I mean the state of the patient sometime previous to the overwhelming assault. I am convinced, from ample experience, that such prostration of the vital powers never occurred unpreceded by a certain portentous state of the whole body, which was well sketched by Hippocrates, and elegantly portrayed by Celsus, though not connected with an antecedent and succeeding series. In their times brevity arose from necessity; their works, therefore, are not much more than note-books to instruct the instructed only. The signs of this state are concealed from the unwary, under the alluring mask of felicitous health; so that when there are unusual vigour, alacrity, and activity of mind and body; when the eyes sparkle with unwonted brilliancy, and the whole complexion is conspicuously improved; when every faculty and every function is exercised with a facility and a power that the person has seldom or never experienced, let him beware; he is on the brink of a precipice; and if an epidemic prevails, it has already seized him; his fall will be sudden and tremendous, in proportion to the exaltation which he enjoyed. This is the first stage of *the epidemic fever*, inadvertently called *the Cholera.*

The stage just described precedes those of collapse and re-action, the first and second of Drs. Russell and Barry, and *if that were skilfully managed, these would seldom supervene.* Attention to it ought to be insisted on the more earnestly, that it deceives under the appearance of the best health; and for another reason still more urgent, that during this stage alone can we expect to preserve, along with life, every organ uninjured. Here all the functions, all the organs, are exerting in unison to an intensity that no constitution can long sustain. If, during this excitement, which is that of real inflammation, the fuel of ardent liquors be added, who does not see the inevitable consequence? This explains why the intemperate are the most certain victims; though it is not so much habitual imprudence, as indulgence at this critical juncture, which hastens and ensures the fatal catastrophe, otherwise exemption would have blessed the natives of Hindostan, the most sober and moderate people on the face of the earth. They are not, however, without their reward, since all of them, who escape the extreme danger, recover much more rapidly and completely than their European lords.

It may seem extraordinary, but it is not the less true, *that to the total neglect of this stage*, or *to the not being aware of its existence, is to be ascribed the appalling mortality throughout the Indian and Russian empires.*

Let it be remarked, that this, as well as the other stages, differs in different individuals; it sometimes assumes the unequivocal characters of inflammatory fever, with quick and strong pulse, and apoplectic torpor.

It is truly curious to observe how constrained the mind is by preconceived notions. Medical men were so fully persuaded of *the collapse* being the commencement of Cholera, that occasionally, when the inflammatory state obtruded itself upon their notice, they spurned at it; nor would they believe that it belonged to the disease, they rejected it also, because "these are precisely the cases which yield most certainly and readily to our remedial means." Of this lamentable infatuation, and of the unconscious sophistry by which they evaded the lessons of nature, the documents before me are replete with examples. Though this letter is already long, you will excuse another extract from the "Official Report of the Epidemic Cholera, as it appeared in the territories subject to the Presidency of Fort St. George."

"Of all the symptoms of cholera, none is so invariably present, none indeed so truly essential and diagnostic, as the immediate sinking of the circulation. It must nevertheless be admitted, that, where instant remedial measures have been successfully practised, this symptom may not have developed itself; and that there are even cases, where an excited vascular action has been observed to accompany the first movements of the system in cholera. Some intelligent practitioners have entertained doubts whether such cases belong indeed to this disease; and there seems reason to imagine that those inflammatory affections with spasm, known in this country, and alluded to in several reports, may, in some instances, have been mistaken for it. It is farther to be remembered that these are precisely the cases which yield most certainly and readily to our remedial means: and it consequently follows, that a medical man can seldom have the opportunity of observing whether or not this form of cholera will degenerate into the low stage. There is, however, direct evidence in support of the fact, that they have so degenerated, and gone on to a fatal termination."

Thus chance, or rather Providence, presented them with a gift, the proper use of which would have stripped this disease of all its terrors; but to foster a bauble, a paltry conceit, they threw away a pearl of inestimable value.

To conclude, Sir Henry, in this letter I make no sacrifice to complaisance; for, divested of personality or animosity, it is a rule with me, where the fate of my fellow-creatures is involved, to commend and to condemn with equal freedom. My design was to give a more practical application, than has yet been done, to the pathology of the Indian Cholera, to shew, that the succession of its symptoms is more extensive than it is generally understood to be: *above all, to point out the signs of its commencement, which, when known, will enable us to act so as to render this frightful pestilence comparatively harmless.* I therefore request you to submit these observations, hastily drawn up, though not hastily imagined, to the consideration of the Board, and entreat them to re-investigate, and scrupulously, all the facts and circumstances relative to the Cholera, that they may arrive at a definite judgment concerning its nature, and fixed principles to govern its treatment; which, banishing the dread of empiricism, will inspire the nation with confidence; and though the inhabitants of the United Kingdom may never require the aid of their labours, yet the countless millions in Asia, where this malady always exists sporadically, and so often rages epidemically, will owe them an inextinguishable debt of gratitude. Happy if I should be of any service in such an undertaking!

I have the honour to be,

Yours obediently,

Edinburgh, 15, Duke-street, JAMES SANDERS.

November, 1831.

---

III.

### CHOLERA MORBUS.

Dr. Brown, of Sunderland, to Dr. James Johnson, and Dr. Alexander Tweedie, Physician to the London Fever Hospital.

*Sunderland, Nov. 10th*, 1831.

Gentlemen—The first question asked here, and which probably may be put in London, is, "Is this the Continental disease?" The question is an ambiguous one, and ought not to receive a direct answer; for it may mean any one of these three things, viz:—1st. Do its symptoms correspond with those of the Continental disease? 2d. Has it been imported from the Continent? 3d. Does it, in its diffusion, obey the same laws as the Continental disease? In the first sense of the question, I would answer it in the affirmative; in the second, *decidedly in the negative;* whilst, in the third, my answer would be doubtful, for the laws which the Continental disease obey, in its diffusion, are not as yet fully ascertained.

Need I examine formally the question of its importation, and refute the story circulated through the newspapers of certain ships which lay above our bridge, and communicated the disease to the town? Those ships came from places where cholera did not exist at the time of their departure—most of them from Holland, where it has not yet appeared—their crews were, and had been, in perfect health; and the disease *first* manifested itself in a part of the town *two miles distant from where they were lying.* If there have been other modes in which disease may have been communicated from the Continent, I know not of them. One man, a pilot, was attacked some time ago; he had been on board a vessel, which had recently come in ballast from performing quarantine; but at the same time, cases identical in character were taking place in individuals, who had had no communication with shipping; and assuredly a ship with a healthy crew, in ballast, and which had performed the regulated quarantine, was not a probable source of the disease with which this man was attacked, and under which he succumbed.

The importation doctrine is here—where we must be supposed to be the most competent judges of a matter not of opinion but of fact—so generally abandoned, that I shall bestow no more pains on its refutation; but proceed to give as concise a sketch as I am able, of what we may designate by the antiquated term, the medical constitution of the latter part of the present year, and endeavour to show by what insensible gradations our autumnal disease has passed into the intense form which has produced such lamentable consternation throughout the empire.

Early in the month of *August*, Cholera appeared and speedily became very prevalent. It ranged in all degrees of intensity, from slight bilious attacks, to cases attended with violent spasm, coldness, collapse, almost, (if not complete) arrest of the circulation, *white discharges*, *suppression of urine*, *and*, *in short*, *all the symptoms ascribed by observers to the Asiatic and Continental diseases*. Of these more intense cases, several were fatal, some of them within *twelve hours*; whilst others narrowly escaped by prompt and skilful medical assistance. Such cases occurred in situations remote from each other; some of them several miles *inland*—one, for instance, and that a fatal one, in a female living in the village of Boldon, five miles in the interior, and remote from the river. Others of the agricultural population suffered in various situations; some certainly near the river, but there were no ships in it at the time, which had come from suspected places.

On the abatement of the heat, Cholera became less general; but did not totally cease, cases continuing to recur at intervals, some fatal, others of great intensity, but terminating favourably; whilst the prevailing gastric and intestinal constitution was marked by the frequent occurrence of cases of fever, commencing with vomiting and purging of matters variously coloured—in short, by symptoms of cholera; and this state of things continued till the almost simultaneous occurrence of four deaths from Cholera, on the 31st ult. and 1st inst., excited general alarm. For what has subsequently occurred, the reports to the Board of Health must be referred to.

Whilst matters are thus proceeding in Sunderland and its immediate vicinity, information I have received from various quarters, and through various channels, leaves no doubt on my mind, that a similar train of events has been in progress generally throughout the north-eastern division of the kingdom—the same prevalence of fever, of which the initiatory stage is marked by vomiting and purging—the same occurrence of fatal cases of Cholera, since the season of heat and fruit has passed; but, so far as I know, the same prevalence of the intense forms of the disease has not been manifested elsewhere as here; yet this difference in degree does not, I imagine, make our state *essentially* different. What is our fate to-day may be that of others to-morrow. A fortnight ago we were no worse than our neighbours.

In this Sketch of the general progress of disease, during the Autumn, I have classed together things which, because differing in name, are by many supposed to be *essentially* different—cholera and fever. The relation between these two genera of disease, is as close as possible. If a man is attacked with vomiting, purging, and collapse, and succumbs to these symptoms, he is said to die of cholera; if the disorder of the system is eliminated, as it were, by the discharges from the intestinal canal, he is said to recover from it; but if neither of these events occur, his state becomes one of fever, not distinguishable from that affection unpreceded by choleric symptoms. The first case which Dr. Daun saw, on his arrival here, he said he could not distinguish from typhus—the discharges had ceased, and it had passed into the febrile state. I pointed out this relation between ordinary cholera and fever in the Essays published three years ago, and was not a little surprised to see my observation quoted in the *Courier* newspaper of the 7th inst. Am I then to regard the disorder I now witness here, as something not resulting from indigenous causes—as in no way connected with the progression of disease I have remarked—but as suddenly thrown in and totally foreign from that to which, notwithstanding, it bears so striking a resemblance? I own I cannot so far shut my eyes to facts, or fail to exert my reason in drawing legitimate inferences from them. If I now visit the houses of the opulent and comfortable, I find, as heretofore, bilious attacks easily allayed, or febrile disorder connected with them;—in the hovels of the indigent, I find a disease characterised by the symptoms of the epidemic cholera of the Continent, so far as I can judge of them by the reports of others. The rational inference from this appears to be, that both result from the same epidemic influence modified in its operation by the different circumstances in which the two classes of persons are placed.

In thus stating my belief, that this disease is but the effect of a general epidemic influence, I wish it to be distinctly understood, that I have seen nothing in its progress that has led me to conclude that contagion is instrumental in its diffusion. One or two *primâ facie* cases of infection have presented themselves; but a little scrutiny has shown me their fallacy, This branch of my subject, if it appear necessary, I will resume at a future opportunity.

It must be evident from the opinion just expressed, and the general tenor of my communication, that I should deem all restrictive measures on internal commerce as useless in a preventive point of view, as they have already proved on the Continent, whilst, on many grounds, their impolicy is obvious.

I remain, my dear Sirs,
Your very obedient servant,
J. BROWN, M.D.

---

### IV.

### TORTI ON PERNICIOUS FEVERS.

Dr. Negri, a very intelligent and erudite Italian physician, residing in London, has published two letters in the Medical Gazette, in which he has shown the great resemblance, if not identity, between the malignant cholera and the pernicious fevers described by Torti, more than 120 years ago.

"Speaking of the character of those fevers, Torti says, 'the pernicious intermittent, more especially that wearing the tertian form, kills about the beginning of the paroxysm, when it is accompanied with violent bilious vomiting and purging of bilious humours, equally vicious both in quality and quantity, being sometimes clear, at others coloured, and occasionally of inspissated greenish bile; to which vomiting and purging are added, hiccup, a hoarse sonorous voice, hollowness of the eyes, pain of stomach, small sweat upon the forehead, weak pulse, and cold or livid extremities—in one word, all the symptoms which usually mark *cholera morbus;* from which, however, this, as it were, *choleric affection*, is to be distinguished, since it is a *mere symptom* of the fever, the period of which it follows, *as a shadow does a body.*'"

Torti describes a "*febris perniciosa cholerica*," in which the patient becomes nearly exhausted, "universally chilled, lies supine, with a pulse almost abolished, sunken eyes, and difficult breathing." Dr. Negri also quotes from Mercatus, physician to the King of Spain, who describes a pernicious tertian, presenting the same symptoms as cholera, and frequently lapsing into a pernicious fever. The following passage from Morton, quoted by Dr. Negri, will be read with interest at the present moment.

"Among the innumerable symptoms attending these fevers, there is none which may not rise to a great height, endangering the life of the patient, so that *typhus fever* (masked in its stages of cold, heat, and sweating) supervenes, rendering it impossible to be distinguished by the *urine*, *temperature*, *pulse*, or indeed any other means; but, concealed under the appearance of cold, vomiting, diarrhœa, *cholera morbus*, colic, or other disease, not unfrequently misleading the physician."

Torti, as well as Morton, exhibited bark as early as possible, and in large quantities, and this practice is recommended by Dr. Negri, from experience of its good effects in the malarious fevers of Italy. Dr. Negri comes to the conclusion "that the *malignant cholera of our days* belongs to the same class of diseases which was seen by Mercatus in Spain, Torti in Italy, and Morton in England." He suggests the administration of bark in large doses, and early in the disease.

The following case from Torti presents a complete picture of the Sunderland cholera.

"When I reached the patient, he had been several hours labouring under the disease. I found him universally cold as marble, with the pulse altogether, if I may so say, absent, breathing laboriously, and having a leaden-coloured countenance. There was some torpor, but no confusion of intellect *(he never mentioned delirium)* and his urine was secreted in a small quantity. I prescribed the bark in large doses. A gentle heat soon pervaded his entire frame; the pulse gradually returned; the respiration became natural; the face lost its leaden hue; the urine was secreted in its ordinary quantity, and in three days he was quite recovered."

### THE PRESENT EPIDEMIC.

The more we reflect on the subject under consideration, the more we are led to think that the cholera now in England is an epidemic *choleric fever*, which has shewn itself, in various modifications, throughout the whole of the Summer, Autumn, and up to the present time. An immense proportion of our population is insusceptible, or nearly so, of the poison—a smaller portion is susceptible of it in various grades of power, in the following ways, according to our observations. In the lowest grade, the epidemic poison merely creates some uneasiness in the line of the primæ viæ, more especially in the evening and during the night, when the chyme and fæces are passing along the intestines, but without any increase of the alvine evacuations. In the next grade, to the above uneasy sensations are added twitches of griping, and one or two additional evacuations daily, of a looser consistence than is habitual to the individual. In the third grade, there is a mild diarrhœa added to the uncomfortable sensations and occasional gripings, the motions being feculent, but thin and accompanied by much flatulence, and some discharge of intestinal mucus. In a fourth degree, the bowel-complaint assumes a severe form. The individual complains that his food runs through him—he loses appetite—gets thinner—and looks ill. In this degree, medical assistance is called in, whereas, in the preceding grades, the patients generally have recourse to domestic remedies. In a fifth grade, there is an approach to dysentery, and some pyrexia attends the complaint. The motions are mucous, watery, pale, or bilious, according to the idiosyncrasy of the individual. In a sixth grade, the disorder assumes the form of common autumnal cholera, there being vomiting as well as purging; the ejected matters being first the common contents of the primæ viæ—then watery and mucous—and lastly bilious. In the seventh or highest grade, we have, especially among the drunken and debauched, and in filthy, crowded, and malarious localities, the intense form of the epidemic, exhibiting the symptoms of the Sunderland and Newcastle disease—first the cold, blue and non-secretive stage—and then, if the patient survives this stage, a typhous fever.

We appeal to our practical brethren, throughout England, whether this is not a representation drawn from nature and facts, instead of closet meditations. The susceptibility to this intense grade of the reigning epidemic, is, taking the whole population of the country into calculation, limited to an extremely small portion of society. It is limited, generally speaking (for exceptions will be found to all rules) to those who are most susceptible to any reigning malady, from the state of their own constitutions and habits—and who live in places the most injurious to health. All the severe grades, however, might be conveniently and practically grouped under four forms of the epidemic malady, namely, 1st form, gastro-intestinal malaise—2d form, diarrhœa—3d form, common cholera—and 4th form, ASPHYXIAL FEVER, or, as it is commonly called, ASIATIC CHOLERA. We conceive that the cause of these four modifications of the same disease, is to be sought in the predisposition of the individual, and the degree of intensity in the poison. In the crowded and filthy hovels of the poor and the dissolute, both predisposition and poison will be found in their intensest degrees; and there the malady will be rapid in its march, and fatal in its termination. In the middling and upper classes of society, the intensity of the poison can seldom or ever be great, and the severity of the epidemic will always be in proportion to the predisposition of the individual. On this account, the upper classes have the means of preservation very much in their own power.

Finally, after the most impartial deliberations of days, weeks, months, and years—we are unable to satisfy our minds, as to the mode of propagation. Looking to individuals, the disease appears to be incommunicable. Looking to the progress of the malady, from the banks of the Ganges to the banks of the Wear and the Tyne, the germs or cause of the disease appear to be carried along, in a North-west direction; but whether by persons, by goods, or by some inscrutable state of earth or air, we are unable to imagine—or at least to decide. We would advise our brethren, therefore, to lay aside prepossessions and theories—to observe accurately—to weigh facts impartially—to be slow in drawing conclusions—and to exhibit less acrimony to each other, when they differ in opinion. By this philosophical conduct they will promote more effectually the cause of medical science, and expose themselves less to the censure or the ridicule of the non-professional world.

### V.

EXTRACTS FROM MEDICAL REPORTS, FOUNDED ON ACTUAL OBSERVATION, AND COMMUNICATED TO THE GOVERNMENT, ON THE CHOLERA MORBUS WHICH PREVAILED AT DANZIG BETWEEN THE END OF MAY AND FIRST PART OF SEPTEMBER, 1831. By *John Hamett*, M.D., &c. &c. R.N. One of the late British Medical Commission at Danzig, &c., in the North of Europe.*

### *Circumstantial Report on the first Appearance and subsequent Spread of Cholera at Danzig.*

IT remains a problem to this day, in what manner the Cholera Morbus originated in and about Danzig: certainly it is not proved to have been brought hither from Russia or Poland by men or merchandise; because no ship had arrived at Danzig from any Russian port previous to its appearance, and the intercourse with Poland had ceased since the beginning of the Winter. The first symptoms of cholera shewed themselves indeed in such a peculiar manner as almost to exclude even the suspicion of its importation; and it is reasonable to conclude, that the disease originated here in some manner that has, as yet, not been explained.† This is corroborated by the statements of several physicians, viz., that cases similar to cholera had been observed previous to the arrival of any vessel from Russia; and that the weather had been so remarkably unsettled since the commencement of Spring, that malignant diseases might be reasonably anticipated.

In accordance with this expectation, the clergy of the city received orders on the 11th of May to report twice a day to the town physician the names of those who died; the physicians to announce every case of suspicious sickness or death; the police officers in the different districts to have a watchful eye on every case of sickness, to procure correct information of the same, and to give daily, and, if necessary, instant notice of them; while the magistrates were requested to render the police officers due assistance through the inspectors of the district, and the wardens of the poor; and in general every thing had been prepared to obtain speedy information of all suspicious cases, and to make the necessary arrangements according to circumstances.

The two first acknowledged cases of epidemic cholera occurred on the 27th of May, in the Neufahrwasser, or harbour-canal, one German mile from Danzig, in two mud-barges used for keeping that canal deep; the two next on the 28th, besides a suspicious case; but it was not until the 29th that these cases, particularly the latter, were discovered; and on the 30th, that the public authorities announced the existence of the epidemic accordingly.

On accurate inquiry, however, two or three cases of the malady appeared to have occurred in the town of Danzig on the 28th, in two quite different parts; since which it has been ascertained that isolated cases, similar to cholera, were witnessed at an earlier period than the 27th.

The discovery of the first appearance of the epidemic on the 29th did not take place in the harbour-canal where it had broken out, but in three or four villages nor far from each other in the Danzig Nehrung, in the following manner:—

---

* After the last sheet of our Journal was worked off, we happened to see the following document in the hands of a friend, and having borrowed it, we take upon ourselves the responsibility and blame, if blame there be, to make it public, for the good of science and society at large.—ED.

† I have with great pains sought for its origin in the different physical states of the atmosphere for the last six years, in connexion with soil, localities, &c.

The physician of the Danzig district, Dr. Lenz, was informed, on the 29th of May, that, of four labourers who had fallen sick in the mud-barges in Neufahrwasser, and had been conveyed to their homes in the villages of Nickelswalde, Krohnenhoff, Einlage, and Schnackenburg, in the Danzig Nehrung, three had died suddenly from some suspicious sickness, and one was still alive. He found symptoms of Indian cholera in this patient, and judged the above three had died of cholera. He reported these cases the same evening to the Regency; and in a conference which took place at ten o'clock the same night with the chief president, Rothé, it was resolved not only to isolate immediately the said villages, but also to investigate the particulars of the mud-barges. This investigation was accordingly made the next morning by some of the police, and the city physician, Dr. Mathy, when the following particulars were ascertained:—

The labourers in the two mud-barges had been in active employment since the 16th of May on the six working days of the week. The two barges had, under the direction of their respective overseers, Gutziel and Wolff, one thirty-six, the other thirty-four men; all of whom, on the arrival of the Commissaries, on the 30th of May, at eight o'clock in the morning, had been hard at work since half past four; and except the above-mentioned four, and one suspicious case, were all found in good health.

With respect to the labourers in the mud-barges, it will be proper to state, that by far the greater number of them are inhabitants of the Danzig Nehrung, belonging to the Danzig Landrath district; and only few of them live in the suburbs of Neufahrwasser; that the former go regularly every Monday morning to the mud-barges, in which they literally live, remain there during the week, and return on Saturday with their weekly wages to their families. Their food consists chiefly of potatoes, groats, bacon, and sometimes flour dumplings. Of these they take a sufficient quantity to last the week; and are, therefore, seldom under the necessity of leaving the barges. As, therefore, they had seldom any communication with other people; and as in the villages in the Nehrung, and in the suburbs of Neufahrwasser, as has been stated, no illness had occurred before these individuals fell ill, there was no evidence of contagion in these instances.

No other case of cholera occurred in the mud-barges, besides these; the mud-barges, however, soon ceased to work after this.

In all the vessels lying in the harbour, no case of fatal sickness took place, until the 30th of May, neither among the numerous labourers employed in building the pier, nor in the other establishments at the harbour.

On board 110 ships from Russian ports, laden with provisions, &c. for the Russian armies in Poland, which arrived at Danzig between the 30th of May and 17th of August, Captain Brandt only, as it is presumed, died of cholera on the 31st of May; in the Contumace Establishment at Bresen, only two seamen died of it; at the unloading of the cargoes of these ships in the roads and harbour, and on the Vistula, no one died.

The first vessel from Russian ports was the Monna, Brandt master, direct from Riga. This vessel arrived in Danzig roads early in the morning of the 30th of May, three days after the first appearance of the epidemic, and was allowed to enter the harbour the same forenoon, having a clean bill of health from the Prussian Consul at Riga. Mr. Gibsone, the British Consul in Danzig, ascertained that he had not been well while on his passage, and that he died on the 31st of May, and not on the 1st of June, as stated in the General List. This is only a presumed case of cholera, he not having been visited by any physician in his last illness. His two sons and the mate of the Monna attended on him, and they, as well as the crew and pilot, escaped the disease.

That ships from Russian ports did not arrive at Danzig before the 30th of May is ascertained by the list of arrivals here, which lies open to every one's inspection; at the same time it should be mentioned, that the earliest information of the cholera having appeared at Riga, was received on the first of June by Kitskats, the ship's broker, who had it from the Prussian Consul, General Woehrmann, at Riga, in a letter dated $\frac{14}{26}$ May.

On the 30th of May cholera appeared not only in the town, but also in the suburb Schlapke belonging to Schidlitz: thus, at the same time in two quite different parts of Danzig. It attacked several individuals in Eimermacherhoff, Rambau, and Seigen, three streets adjacent to each other, not far from the principal ramparts of the fortress, and Mottlau guard-house, which is situated on the confluence of the Mottlau and Radaune streams, into which all the dirt and filth of the low and old town in particular are conveyed. The Mottlau, which is the larger stream, is lost in the Vistula at about 2000 paces from its place of junction with the Radaune. The above mentioned streets are in the lowest part of the city, and were in March, 1829, quite inundated, the water rising, in several houses, from five to six feet, and in some even to the ceilings on the first floor. The ground there is rather marshy, and intersected by drains for carrying off the dirt and filth. When the Vistula is higher than ordinary, parts of these streets are generally more or less inundated.*

The suburb Schidlitz, distant about a quarter of a German mile from the city, is in a dry and more healthy situation; the houses are more airy, and less contiguous than those of the above named streets, and the manner of subsistence and occupations of the inhabitants of a totally different nature: those of the above-mentioned part of the city gaining their livelihood chiefly by such occupations as relate to shipping, while those of the suburb of Schidlitz and Schlapke are chiefly agricultural or mercantile labourers, and subsist on the produce of their gardens. *Here only one woman fell sick at an early period, and subsequently but few cases appeared.*

It not only appeared, as above stated, in the old town, but continued to spread, without any marked order from personal contact or proximity, in low, damp, and dirty, or close situations, all over the city, among the destitute and poor; who are here, in general, ill-clothed, ill-fed, uncleanly in their persons and dwellings; never wearing flannel next the skin; subsisting chiefly on indigestible and unwholesome food, and in the habit of using pernicious drinks:—habitual drunkards of whatever class have been invariably the victims of this malady. Besides these, it occasionally selected for its objects, in comparatively healthy spots, persons of particular constitutions and habits, in easy circumstances of life, who happened to suffer in their health, in the various ordinary ways from exposure to cold damp air, especially at night; from profuse perspiration suddenly suppressed, the bad effects of conjoined wet and cold, &c. &c., and, above all from recent derangement of the stomach and bowels. The higher and middle classes escaped the disease; a few excepted, who brought it on by a want of common care of themselves, or who became incidentally predisposed to it, as shown in the Police Reports, and the interesting communications to me from Mr. Gibsone, the British Consul in Danzig; they not having been near, or at least ascertained to have been near, infected persons.

In proof of these statements, between the 28th of May and the 23d of July inclusive, 835 persons, consisting almost entirely of the wretched and unhealthy poor, were attacked with cholera, of whom only 195 recovered, according to the General List, making the amount of deaths during that period 640. During this period of eight weeks, notwithstanding that 2000 inhabitants of the dwellings of these sick were shut up for twenty-one days, subject to the bad effects of fear, want of exercise, and fresh air, though indeed having the essential advantage of being well fed, only 188 persons fell sick in 108 houses, in each of which,

---

* An inundation, unexampled in the memory of man, took place in this month and year, and laid twenty German square miles of the immense plain, or valley of the Vistula, as it is called, under water.

at the same, or at different periods, there was more than one patient;* and in these 188 patients, a probable or predisposing cause to the disease has been officially reported in the Separate General List.

In a population of between 70,000 and 72,000 in Danzig and its immediate district, the whole mortality from cholera was officially reported to have been 1028 of both sexes and all ages, coinciding in all particulars of the disease with the above-mentioned 640, between the 30th of May and 8th of September. The numbers actually taken ill of the disease I have been unable to ascertain. The physicians in Danzig, as I repeatedly stated in my reports home, did not report all affected with cholera.

As it is of importance to know the proportion of its victims of both sexes, and at the different decimal periods of life, I beg leave to state, that of these 1028, according to the official report alluded to, 539 were men, 368 women, 59 boys, and 62 girls. Of these, 26 males and 20 females died at different ages under six years.

*Descriptions of the Symptoms of the three Principal Forms of the Epidemic at Danzig; being Extracts from the circumstantial Detail of the Symptoms of it, including its subordinate Features.*

PASSING over subordinate features of the epidemic, I shall limit my descriptions here to the three principal forms of it; viz.

1. The rapid and severe cases of fatal cholera.
2. The protracted cases of fatal cholera; and
3. Those less severe, which proved favourable.

1. In most rapid and severe cases of fatal cholera, the patient was suddenly seized with sickness or pain at stomach, occasional pain, or feeling of weight and uneasiness in the hypochondria, the right hypochondrium especially; giddiness, prostration, great thirst and craving for cold drinks, a cold sweat that quickly became colliquative and clammy; at times coldness alone, at others coldness and dampishness of the body, but never with shivering; the pulse was frequent but not hard, and soon became exceedingly reduced; the hands and features somewhat shrunk: the tongue was foul, unnaturally moist, and occasionally tremulous; the voice subdued; the eyes heavy and suffused, and the sight dim. These primary symptoms were in general either accompanied, or immediately followed, by retching and vomiting, and a peculiar watery diarrhœa, which often, however, proved irregular in the order of attack, occasionally even with respect to each other, and oftentimes severe, in hot, close, and electrical weather especially; griping pains in the abdomen; painful contraction of the muscles at the umbilicus; suppression of the secretion of urine, and occasional pain in the region of the bladder. Cramps in general followed the retching and vomiting, and in most instances invaded the calves of the legs at first; in their attacks of other parts of the extremities, they proved irregular, seizing first the fore-arms, calves and fore-arms, hands and fingers, toes and feet, or hands, feet, and calves, in different instances indiscriminately; occasionally they mounted up the thighs, but seldom attacked the trunk. Men rarely escaped them, women frequently, and children generally.

---

* As in the low parts, particularly of the old town, of Edinburgh, so in the low and the old town of Danzig, the houses are composed of stories, or flats, in each of which one or more families reside. But, unlike the old town of Edinburgh, there is in general here a large privy in each house, which is seldom emptied until it is full, or nearly full. The sewers are, for the most part, made of wood, not close, and prove bad conductors of the dirty fluids thrown into them. Hence the offensive effluvia occasionally so common here at those changes of the weather when the mercury rises in the barometer.

The vomited matter in general consisted of undigested food at first, sometimes partially tinged with yellowish green; of fluid ingesta, also occasionally imbued with greenish coloured matter, and partly of slime and mucus. Often, however, it consisted of undigested food, or of fluid ingesta alone, without being in any wise so imbued. In the retching and vomiting which followed, the fluids taken continued to be rejected with a little greenish coloured matter, with or without more slime or mucus. The dejections were always watery; sometimes as if coloured with feculent matter; in general they were either colourless, somewhat like whey, or had the appearance of rice-water, barley-water, occasionally somewhat dirty, or with an avenaceous sediment, after being shaken in water.

After this first advance of the disease, the following symptoms rapidly supervened; viz. increasing oppression at the heart, and short hurried and laborious breathing, ending in complete oppression and weight at the *præcordia;* tossing of the head about; anxious restlessness depicted, often with terror, in the countenance, which in general was of a dark brown, wan, or leaden hue, according to the complexion; insatiable thirst, with incessant craving for cold drinks, and the voice raucous and depressed. The retching and vomiting, and diarrhœa, with occasional *tormina* and cramps, at first only intermitting at short intervals, subsided either abruptly, or gradually as vital exhaustion advanced; the pulse at the wrist, if not extinct—which it was in most rapid and severe instances—was accelerated to the utmost in frequency, and barely felt; the surface of the body quite cold, damp, and clammy, and the feet and insteps marked with bluish streaks and patches; the tongue cool or cold, and in some instances livid at the tip and edges; breath cool or cold; lips blue; nose sometimes bluish; voice below the breath, or gone; cheeks and eyes now quite sunk; pupils at times partly or completely dilated; eyelids half closed, and encircled with livid rings; the parts of the conjunctivæ exposed being much the same in appearance as after death. Amid this complicated suffering, the patient was not insensible until just before dissolution, which ensued after some faint convulsive sobs, generally within from eighteen to seventy-two, and occasionally within from eight to eighteen hours after the first attack.

2. In the protracted cases of fatal cholera, which have been few in number compared to the rapid cases, the following febrile symptoms have been observed, more or less, in different patients after the indefinite period of the first stage: namely, marked congestion, with pain in the head, deafness, humming noise in the ears, heavy stupor, continual drowsiness, partial ravings; a dark flushed, brownish yellow, squalid or cadaverous countenance; a dark brownish clammy, or furred tongue; dark sordes about the teeth and lips; eyes heavy and suffused, or dry and parched, often with eventual dilatation of one or both pupils; a hot or cold clammy skin; pulse frequent, with febrile action, or very small; with pain or soreness of the abdomen, increased on pressure—and occasional tenesmus. With these symptoms, the excretions, as may be readily conceived, were scanty and vitiated. The stools dark, dark-green,—very fetid, and the urine in general dark coloured. Delirium generally took place in these before death, and they died within from three or four to five or seven days, or later, after the first attack; more generally on the fifth.

These modifications of particular symptoms, bordering on each other, and referring to individual parts, depend, I need scarcely add, not only on differences in constitution, but, in a certain degree, on the mode of treatment at the commencement, and even on the state of the locality in which the patients happened to be placed.

3. In the cases less severe,—and as I have observed,—of less unhealthy persons, in whom the natural powers* of the constitution were calculated to with-

* This is certainly true; and yet a woman, named Eliza Brandt, thirty-six stand the effects of the shock on the system, giddiness, retching and vomiting, watery diarrhœa, occasional griping pains in the abdomen, cramps, occasional painful contraction of the muscles at the umbilicus, thirst, and suppression of the secretion of urine, took place, and proved occasionally severe ; but the congestion in the head, and oppression in the chest were certainly less marked; the pulse, although barely felt, was rarely entirely suppressed ; coldness of the body, the cold, clammy sweat, and other bad symptoms were not marked in any great degree. The leading symptoms gradually, or abruptly disappeared ; and more or less of febrile re-action ensued, generally within from eighteen to twenty-four or thirty-six hours, or more, after the commencement of the disease, about which time hiccups, always a favourable sign, were occasionally noticed, and not before. The exact period of the return of the urine was not certain, being sometimes before, at others after the first appearance of re-action : it was dark or high coloured, voided in small quantities with occasional difficulty, and frequently attended with some pain in the region of the bladder. The return of urine, though an important symptom, was not always decisive of a favourable result ; on the contrary, hiccup, which however was not always observed, almost invariably indicated recovery. The dejections immediately after the commencement of re-action were fluid, scanty, and dark-coloured, as if imbued with blackish feculent matter ; but they very soon became successively brownish, and naturally bilious and feculent. Indeed, in the majority of cases of this description, the secretions and excretions soon got into play, and restoration was more or less rapid.

Partial stupor, with little or no delirium, more commonly occurred in children, and spare aged persons free from previous organic, or general complaint, and gave grounds for a favourable prognosis : they seemed tranquil, and as if naturally asleep. They were in general affected with *œdema* in the feet, and more or less in the legs after convalescence had commenced. *Œdema* also occurred in others after the disease, but not generally. In pregnancy, abortion invariably took place, and was always a critical symptom, death or a favourable change soon following.

From the description above given of the rapid and severe cases of fatal cholera in Danzig, its similarity to the Indian cholera appears manifest ; and from the descriptions of fevers supervening after the first stage, as given in the second and third forms, its deviation from the Indian epidemic, in which those fevers do not generally supervene, also appears evident. The greater severity in general, which has been found of the vomiting, diarrhœa, griping pains in the bowels, and painful contraction of the muscles at the umbilicus, in the epidemic in India, compared to that in Danzig, is easily explained by the well-known influence of the climate in India on the whole system, and digestive canal in particular.

JOHN HAMETT, M.D.

*Extracts from the Pathological Report on fatal Cholera, both rapid and protracted, founded on the Examination of twenty-one Subjects ; the youngest of which was four, and the oldest ninety years of Age, the rest having been of adult and middle Ages.*

Many of the characteristic appearances after death will depend, in a great measure, upon the number of hours elapsed before the body is opened ; the later the examination, the less truly characteristic, so far, are the appearances. Those

---

years of age, affected with tubercles and vomicæ, had, in July last, this less severe form of cholera, which soon gave way to all the hectic symptoms of her complaint.

I have examined were in general opened within, or about, twelve hours after death. Bodies at this season ought, however, to be examined as soon as possible, and always within at least six hours, if it can be done with propriety.

Of all the morbid effects in appearance, which I have observed after death in the bodies of persons who died of cholera in Danzig, the most characteristic, perhaps, has been the great congestion of blood in the sinus venosus and right auricle of the heart, and in the veins throughout the whole body; the next is the invariable contraction of the bladder; and another, which, although not apparently constant after death from this disease, is seldom or never to be met with after death from others—namely, slight spasmodic contractions, or movements, if they may be so called, in the muscular fibres here and there in the body, and more especially in the face and extremities, not only immediately, but some time after dissolution. These resemble Galvanic effects produced in the body after death.

The veins, and right auricle in particular of the heart, were full of black blood; some was always found in the left auricle; while very soft imperfectly coagulated lumps were found either in the right ventricle or within the aorta, either immediately at its commencement, or down below its curvature. These lumps were invariably as black as the blood found in the veins and right auricle; the thoracic aorta uniformly contained some black blood, but was never full, like the veins; the abdominal aorta also contained a little, but very little; the right ventricle had always a small quantity of black blood, the left ventricle a very little. The pericardium seemed more or less flaccid, and very often contained a quantity of dark brown serous fluid. The parietes of the heart in general seemed soft, and I fancied, in a few instances, that those of the left auricle seemed thickened; this, however, remains to be confirmed or refuted by subsequent examinations. I occasionally observed morbid blackish, or bluish, and, in one instance, whitish spots on the external surface of the heart.—The lungs were in general much more bluishly speckled than in most other cases,—almost always collapsed, but dense from black blood—not as in hepatization of the lungs—frothy, black blood freely oosing from incisions made into them. The pleura, in its reflections throughout, from the anterior to the posterior mediastinum, and over the upper surface of the diaphragm, seemed in general of a dark dull red. The trachea, bronchia, and larynx contained a little frothy mucus, and were otherwise wet with a compound of serous and clammy fluid; but the internal mucous surface exhibited no vascular appearance. In general there was a considerable quantity of clammy, serous fluid found effused in the chest; all was wet, exceedingly soft and clammy, more so than I have been used to see after death from other diseases. The vena azygos was invariably full of black blood. The thoracic duct was in general empty, and seemed natural.

On detaching the calvaria from the dura mater, the latter was, in most instances, spotted all over with the black blood that instantly issued from the torn vessels, especially along the lines of the sutures, where they are most numerous, in the younger subjects particularly. The external surface was mostly of a dark bluish colour, and dry, but clammy feel. The internal surface of the dura mater, and its processes, or continuations, were not marked by any peculiarity, except, perhaps, in the appearances being more opaque, and feeling more clammy than usual. The tunica arachnoidea was in general of a wheyey, glossy colour, and somewhat clammy to the touch. Between this membrane and the pia mater, and more especially in the lower part of the cerebellum, there was occasional effusion or filtration of serous fluid; and in all instances there was considerable effusion of this fluid between the pia mater and the cerebrum and cerebellum both; in most instances it was found in the ventricles, in the fossulæ at the basis of the cranium; and, indeed, wherever this effusion between the tunica arachnoidea and pia mater in parts of the cerebellum, and the pia mater and the brain itself at large, was observed, it was also invariably observed in the same relative situations in the spinal marrow of those bodies in which the spine was examined—which were fifteen in number. In other instances, too, where there was effusion in the brain, we had only to elevate the pelvis and loins in order to see serous fluid issue forth from the spine through the occipital foramen. There was always a considerable quantity of thin black blood in the sinuses, in the inferior more so particularly. In all cases the congestion of black blood in the veins of the pia mater, in the venæ Galeni, and choroid plexuses, was great, accompanied with varicose dilatation of these vessels; and likewise the same relative congestion of black blood in the veins of the pia mater, in the spine, especially in the posterior parts of it, where these vessels being larger and more numerous, varicose dilatation was more conspicuous. The medullary substance of the brain seemed in some instances much softer than usual, but it might have been owing, in part, to the interval elapsed during hot weather between death and the time of examination. In some instances black spots were visible on incisions into the brain; at times, too, the cineritious and medullary substance both seemed relatively altered in appearance as well as consistence.—The state of the spinal marrow corresponded in all cases exactly with that of the brain.

After what has been said and implied of the venous congestion in the brain, spinal marrow, and thorax, it will be readily conceived in the abdomen, in which the large as well as small vessels are still more numerous and varied. The vena cava abdominalis and vena portæ, with the splenic and superior mesenteric trunks, and, in short, all their large tributary branches, invariably contained a considerable quantity of black blood: they seemed at times as if full of it, while the mesenteric veins always exhibited a characteristic black or bluish arborescent appearance throughout. The gall-bladder was not only of a deep green externally, but, in some instances, from a deep green to a bottle-green, and occasionally tinged here and there with yellow; and was in general distended, and full, or nearly full of fluid, generally black, and sometimes as if a little of yellow or brownish yellow bile had been mixed up in it. The internal or villous coat of the gall-bladder was in general between a dirty yellow-brown and brownish yellow —in a few instances it was a natural bilious yellow. The liver was invariably in a state of *engorgement* from the black blood, which, in all states of it, freely oozed out from the hepatic veins in particular on incisions into its substance: it was in general discoloured, even after sponging the membrane covering it, and I think most in the younger subjects, and those who had not suffered from previous affection of it. The spleen was also in a state of *engorgement*, and of a black purple colour,—and this independently of any alteration in its structure as referrible to other morbid states. The kidneys, notwithstanding the suppressed secretion of urine, did not exhibit any peculiar change in general, further than that of venous congestion. The same was observed in the pancreas. It is not easy to say whether the ductus communis choledochus, and immediate biliary vessels were in general contracted or not; sometimes I found greenish or vitiated bile at the opening of it into the duodenum, and sometimes I did not. I often found, in protracted cases particularly, the external parts of the duodenum and colon in contact with the gall-bladder, or near it, completely discoloured with yellow bile.—With respect to the stomach and intestines generally, I cannot say that I observed any effects of the disease beyond what is referrible to congestion of blood in the veins, and what might be attributed to the sedative nature of the disease. The mucous coat of the stomach, in particular, and parts of the colon, seemed, in some instances, soft, as if half macerated; indeed, the intestines generally seemed soft, and as if the internal mucous and villous coat could be separated from the muscular coat. The small intestines, I mean the jejunum and ileum chiefly, were more commonly of a dark dull red, or rather of a dark dull slate colour, on their external peritoneal coat, without any positive vascular appearance; sometimes of a pale slate colour, with vascular injection, or vascular congestion more marked; while, on the internal surface, they did not exhibit the same colour generally,—still, in some instances, there was in some parts a modified appearance of it; while in various parts in others there was a manifest vascular appearance of the internal mucous and villous coat, though by no means corresponding to that externally. Besides the pale slate or leaden colour, and the dark red slate colour, I have observed a vascular dark red also—facts which will account for that tenderness or pain on pressure of the abdomen, so marked in cholera, especially in protracted fatal cases. In one instance of a young woman, who had died of true and very rapid cholera, the general external appearance of the whole of the small intestines was of a pale or light rosaceous hue, while that of the colon was quite pale. The mucous membrane throughout the whole canal was whitish, and as if half macerated.—Whether the brown patches, which are at times observed here and there on the internal surface of the stomach and intestines, are effects of the disease, or of previous chronic inflammation, is in some instances not easy to determine. The stomach and intestines, as might be expected, mechanically retained the last fluid ingesta; for, latterly, what came away, did so involuntarily. There were the remains of former mucus, more or less, throughout the whole digestive canal; and in true, rapid, and fatal cholera, little or no remains of feculent matter, except in its usual receptacles, namely, the commencement of the colon, the cæcum caput, in the transverse arch occasionally over across it, and in the sigmoid flexure, in which, in some instances, scanty portions of it were found. The mucous follicles in the internal membrane of the colon at its commencement, and Peyer's glands in the end of the ileum, were occasionally found in large compact patches, more or less continuous, distinct, elevated and somewhat indurated. Brunner's glands, as they are called, were not so observed in the duodenum. The colon externally, as well as the duodenum, particularly at its upper curvature, was discoloured at the upper part of the ascending portion, and beyond in the greater part of the transverse arch, but in the other parts it was of a pale, or pale lead colour. The peritoneum, in all its detached reflections, was more or less opaque, having lost its shining, glossy colour more than in most other congestive and sedative diseases of the system attended with fever, more even than in the compounds of remittent and intermittent fevers in tropical climates, with marsh miasmata, in which venous congestion is so very notorious.—In protracted fatal cases I occasionally observed chronic discolouration here and there on the internal surface of the stomach and intestines—in some instances of a dark brown, in others of a dark brown red, without being exactly vascular in appearance: at times vascular spots and patches were observed in some parts of the intestines, and the dark brown, and dark brown red in others; they were generally in the colon, the commencement above and below particularly, in the transverse arch, and sigmoid flexure. I observed parts of the colon in a gangrenous state, and chronic inflammation of the whole of the ileum, in one subject.—In several instances the lumbricoid ascarides were found in the intestines.—In some instances the commencement of the thoracic duct or receptaculum chyli seemed quite close and contracted.—The invariable close contraction of the bladder, I have not omitted to mention; it was mostly lined with a little whitish mucus.

John Hamett, M.D

*Statements of the Medical Treatment occasionally pursued by some of the Physicians in Danzig.*

General blood-letting has not been adopted in the Cholera Hospitals, No. 2 and 3, which received the unhealthy poor of all ages, and those with chronic organic affections. Topical bleedings with leeches, however, to the head have been used, particularly in No. 3. In the Military Cholera Hospital, amid the very young men of powerful stamina, who are immediately attended to after the attack, bleeding is occasionally successfully adopted.

It has been found necessary to guard against the indiscriminate use of the hot water and vapour-baths in hot weather after perspiration has broken out, and, above all, in the clammy stage of the disease, and after marked venous congestion has taken place, when it seems to increase the congestion, which is particularly observable in the brain and heart. Either ought, as I have intimated, to be used in the critical moment at the beginning of the disease, or, at farthest, instantly after, if admissible even then. To obviate the determination of blood to the head, cold applications ought to be occasionally applied to it, while the patient is in the bath; such as muriate of ammonia, during its solution, and other very cooling applications. The patient should be most gently and otherwise judiciously placed in the bath with respeet to the gradually inclined position of the body, and due support of the head, neck, and shoulders; and the immersion, or subjection, should be short, merely time sufficient for the positive communication of heat and its effects, and no longer, when he ought to be as gently and judiciously taken out, well wrapt up in hot blankets, promptly laid on a bed, and gently rubbed with warm, dry, coarse, but soft thread towels, all over; and wiped dry as fast as the clammy sweat oozes out. There is much handy and careful personal management requisite in this essential part of the treatment.

The hot vapour-bath has been, latterly, in general laid aside here. At first it was used, when the congestion of blood in the head particularly, and the bad nervous symptoms in general, were found to be greatly increased in consequence. Dr. Baum tried it in three, who all died shortly after it: he afterwards abandoned the use of it.

Imagining, from the exceeding irritability of the stomach and whole intestinal canal, that they were the primary seats of the disease, some gave the magistery of bismuth, formerly particularly recommended by Dr. Odier, and by Carminati of Pavia, and Bonnet, in France, in cholics, diarrhœa, &c., and lately by the Polish physicians in cholera: and others, amid the complication of morbid functions, with the nervous and animal debility almost wholly in view before them, trusted to stimulants in general, and, above all, to the carbonate of ammonia, a solution of phosphorus in the spirit of sulphuric æther, camphor, musk, &c. &c. With respect to the magistery of bismuth, it should be mentioned, that in the dissections of some of those who died after treatment with it, there appeared, according to the testimony of Dr. Baum, great inflammation of the bowels.

The inhalation of oxygen gas was recommended to the Prussian government by Dr. Schlesenger, of Marienburg, and by Sir Anthony Carlisle, I believe, in a letter in the papers to the Lord Chancellor Brougham: it had been carefully tried in two cases by Dr. Baum; both of which, he states, terminated very soon fatally, although there had been apparent grounds for a favourable prognosis in these individual cases.

Draughts of cold water have been given in insatiable thirst, and craving for it—only by one physician however. A patient, whom I had seen drink copiously of cold water in the morning of the 12th July, died the same evening. Thirst, anxiety, oppression of the chest, and weight at the heart, were the prominent distressing symptoms in him.

The medicines* made use of in the Cholera Hospitals in Danzig have been in general, viz:—

1. Stimulants and antispasmodics: as ammonium carbonicum, liquor ammonii succinici, spiritus sulphurico-æthereus (or Hoffman's anodyne liquor), spiritus acetico-æthereus, æther phosphoratus, *i. e.* solutio phosphori in spirit. sulph. æther. gr. vi. in hujus ℥j; tra. ambræ griseæ cum moscho, mistura camphorata, opium, laudanum liquidum Sydenhami; tra. opii simplex, vel thebaica;

---

* Ex Pharmacopœia Borussica omnia.

tra. valerianæ æthereæ, oleum valerianæ, oleum cajeputæ, oleum menth. piperit, oleum animale *Dippelii,* tra. castorei, aqua menth. piperit., and various stomachic infusions, &c. &c.

2. Tonics and stomachics; as elixir aurantiorum compositum, pulvis aromaticus, cortex regius seu cinchona lancifolia, radix calumbæ, radix valerianæ, infusum valerianæ, radix serpentariæ Virginiæ, pulvis Doveri cum cortice cinnamomi, &c. &c.

3. Aperients; as pulvis rhæi, oleum ricini, calomel. &c.

4. Remedial means; as hot bath, 30 R., bleeding from the flexure of the arm, leeches, sinapisms, frictions, and blisters.

The system and scale of diet that have been adopted in the hospitals, it is perhaps unnecessary to mention. JOHN HAMETT, M.D.

### *Extracts from Dr. Hamett's Opinions on the general Mode of Treatment of Cholera.*

IN epidemic cholera here we have, all at once, retching and vomiting, a watery diarrhœa, and sudden prostration of strength, with pending cramps, and fast-approaching venous congestion to obviate; here also are suppression of the secretion of bile, and of the secretion of urine at the same time to obviate, on both of which the healthy state and due circulation of the blood depend; and here also, amid the epidemic, there is necessarily a modification of the disease from difference in age, sex, constitution, and habit. In cholera the three grand functions are each and all deranged; and, therefore, much is to be done in all these at once. It is not this or that partial treatment. The various particular treatment ought to be most prompt*, and, if possible, simultaneous; and, under all the circumstances I have mentioned, must be regulated by the most distressing or prominent symptoms of the whole, on which the rest either depend, or to which they are subordinate.

The first object, accordingly, is to obviate the primary symptoms of the disease as quickly as possible, instantly after the attack has begun. Emetics are occasionally given here, and may, perhaps, be given in the first instance, while crudities are in the stomach,—certainly not after. I never gave, nor did I ever see them given in India: there the influence of climate alone, however, proved a most powerful emetic; but here the irritability of the stomach is not near so great as it is in India; and they leave less aggravated effects after the further vomiting excited by them is over. When admissible, the very mildest certainly ought to be given.

If there are no crudities in the stomach, or the patient is not taken ill after meals, a little of recent aromatic confection and calomel in the dose of from gr. iii. vel iv. ad gr. viii. vel x.†, mixed up with proportional quantities of laudanum, are to be immediately given, and followed up by regulated mixed doses of the compound tincture of cinnamon, or tincture of capsicum, and the spirit of

---

* Indeed, that the disease ought not to be suffered to advance one single step, if possible, without being instantly obviated, is seen from the rapid march of the deadly symptoms afterwards in my descriptions of them.

† These two last I mention from my past experience in India only, and not at all from what I have observed here. I have reason to believe, that I arrested cholera in a few instances by that very dose and warm stimulating drinks. I did not even put them on the list, for they were well in less than twelve hours after: and even here they may be given to men. The average dose is gr. v. ad gr. viii. Calomel is but little used in Germany and France, where the people are certainly less bilious than in England; but the English practitioners, above all, know its virtues, and when indicated.

any of the æthers, or of liquor ammon. subcarbonat. vel, &c., with, if indicated, some drops more of laudanum, in some appropriate vehicle; and again by powerful, permanent, and grateful stimulants as well: namely, established, strong cordials, occasionally good brandy itself, or, what is still better in most cases, proportional quantities of very warm brandy and water, occasionally repeated at intervals; or warm old best Madeira, sherry, or port itself, diluted, or without dilution; and a little of the concentrated solution of ginger in spirit, or Oxley's essence of ginger, or a few drops of any of the essential oils, may be added occasionally to these last.

The administration of these stimulants in all cases, their modified doses, and their occasional repetition, must, I need scarcely add, depend in a great measure on the previous particular susceptibility of the patient at the time, and on the consequent effects produced by them, as well as on the degree of the symptoms they are intended to obviate.

Calomel, in modified doses, however, ought in my opinion to be duly repeated at proper intervals at first, according to the symptoms, or consequent effects produced by it, when it should be modified in its administration, as I shall soon have occasion to explain. In India, I have remembered, it did not increase the irritability of the stomach; on the contrary, it rather tended to allay it in the first instance.

Should the retching and vomiting, and diarrhœa, nevertheless, continue, warm starchy *enemata*, with a little laudanum, ought to be administered, and occasionally repeated.

The hot bath ought, in my opinion, to be used in the way I have mentioned, and by all means quickly at first, especially when the skin is cold, or not suffused with a cold clammy sweat, with dry, warm frictions after; otherwise it is best to dry-rub the body all over with hot cloths, to apply afterwards incessantly hot flannels, and keep dry-rubbing the body gently as fast as the sweat oozes out, and with warm or hot flannels occasionally after: flannels besides, meanwhile absorb all the sweat. Various other handy means of promoting local heat in the trunk and extremities ought to be also resorted to, I need not mention the necessity of duly regulating the temperature of the room, and guarding against the great change to the patient, by removing him well wrapped up in very warm blankets from the bath into his bed.

Of the benefit to be derived from the use of the hot vapour bath, I cannot speak from my own observation here. Dr. Sinogowitz lauds it*, while Dr. Baum decries it; but where heat should be communicated quickly, I think the hot bath is better. At any rate, that time which is so precious here, is lost while waiting for it. In systematic medical institutions, however, it ought by all means to be ready at hand, and tried;—and I need not say how very indispensable, dexterous, as well as careful and tender personal management, and above all keeping the head† free from the vapour, are in the use of this bath.

Stimulating embrocations may be occasionally and beneficially used in the first instance, but only at short intervals for obvious reasons here.

Cholera is, perhaps, the only disease in which bleeding and stimulants are both admissible at the same time on just principles, as well as from the obvious benefit derived from their use. Bleeding ought certainly to be occasionally adopted in the commencement of the disease, especially in young and otherwise stout or healthy subjects, on the principle I formerly mentioned; and even occasionally after re-action, when the state of the pulse and general congestion indicate it.

---

* I saw the hot bath only used in the military Cholera Hospital myself.

† I said in a former report, that the heat of the bath increased the volume of the blood in the head: it may not do so in healthy blood to any degree; in cholera, however, I am persuaded it does; besides here the blood is notoriously altered.

Shaving the head, although I have never seen it done here, and keeping it bathed or sponged with cold applications, ought to be adopted where venous congestion so positively indicates it. Leeches ought to be applied to the temples and nape of the neck in marked congestion ; blisters to the nape of the neck also—and, it would seem, in some cases, even to the epigastrium also, and abdomen especially at first ; otherwise sinapisms in general to these last, as well as to the parts of the extremities most affected with cramps ; and, blisters ought in my opinion, under certain indicating symptoms, to be applied, as well as frictions, to the region of the heart. By obviating congestion of blood in the brain, that in the spine is obviated proportionally.

Mercurial frictions* to the hollows of both thighs, and to the inner parts of both arms, ought, in my opinion, to be occasionally adopted, and that quickly, when mercurials by the mouth prove ineffectual especially.

Antispasmodics, as they are called, properly modified, should be duly or occasionally administered in the first stage or state of the disease.

The furred tongue, in the second stage, as well as the quality of the dejections in all stages ; and the state of the intestines, liver, and gall-bladder after death, and indeed of the spleen as an ulterior retrograde effect, when we see that the current of the vena portæ is stopped from the suppressed secretion of bile, indicate the necessity of promoting biliary secretion and alvine excretion ; and when the retching and vomiting have at all abated, they ought to be promoted as quickly as possible. This may be done, or at least attempted, in the weakest state, and is not incompatible either with the administration of antispasmodics, or regular tonics ; either of which can be administered at the same time.

Combinations (to which I alluded) of calomel and the sulphate of quinine, with or without opium, seem to be indicated, and I think ought to be administered. There is really here some remote analogy between the malignant remittents of marshy places within the tropics and cholera, in respect to congestion of the blood, the absence of biliary secretion often, and great prostration of strength ; and like cholera too, owing to a modification of infectious *miasmata* in climate, and like it also depending a great deal on constitution and habit. The use of diaphoretics, so occasionally useful in protracted fevers, does not seem to be indicated here at present ; and of all these perhaps the *Pulvis Jacobi* in small doses is the most admissible, by reason of its promoting the effects of the mercury. Our unquestionable object here is to excite the liver and absorbents, both ways, by the lymphatics and lacteals, into action, and meanwhile the secretion of the urine itself so dependent as it is on their due action, and to restore the system from its sedative and exhausted state. Mercury with antispasmodics or regular tonics are consequently indicated throughout the disease.

The local treatment I have particularized. The tonics may be certainly varied.

The administration of mild stomachic purgatives, or aperients, should be persisted in, according to the state of the alvine excretions, throughout the whole of the disease, and occasionally followed up by that most efficient and valuable purgative, castor oil.—Salts, in my opinion, are pernicious in the beginning of the diseases, and even objectionable in the latter part ; if given in the febrile state, they ought to be administered in half, or rather the third part of the common dose, and combined with aromatic confection and rhubarb, both to make up the deficiency, and also to modify them. The purgatives, after certain allowable intervals, ought to be followed up by some grateful hypnotic, after their operation is well over : and here the pulvis Doveri seems to be occasionally indicated, as well as other anodynes.

---

* Dr. Baum has assured me that he tried them in the case of a young woman with marked success.

The chances are, I am persuaded in my own mind, that where the treatment is adopted in the way I have mentioned, those marked eventual symptoms of heavy stupor, &c. &c. which I have so minutely described in the protracted cases of fatal cholera, would not ensue. These symptoms are, evidently, the consequences, in a very great degree, of the protracted suppression of the secretion of bile and urine, in the first instance, and which, as I have stated, would have been obviated by the Indian plan of treatment, under the modifications I have mentioned.

I need not enlarge upon the treatment in this stage of the disease; it is plainly indicated;—in the first, and commencement of the middle part of it, amid such a complication of distressing symptoms, only lies the difficulty. However, in this stage, or, strictly speaking, this modified state of the disease, the treatment evidently ought to be with a view of getting the scretions and excretions into play, and duly supporting the patient meanwhile; the former by alteratives, and occasional stomachic, or grateful purgatives or laxatives; and the latter by tonics. Calomel combined with antimonials, or with ipecacuanha, and rhubarb; or the efficient blue-pill combined with these last, ought to be now given here; sulphate of quinine,—interchanging it meanwhile with any of the established bitter infusions, modified by their corresponding tinctures; wine occasionally now also, &c. &c.; and last of all two or three tepid baths.

The particular diet and drinks throughout the disease, I have no occasion to mention. JOHN HAMETT, M.D.

*Extracts—On the Question of Contagion of Cholera at Danzig, considered solely from Facts.*

THE following statements founded on the experience of the physicians in charge of the different cholera hospitals in Danzig, and of physicians, who at the same time attended cholera patients in their dwellings, and patients affected with other diseases, will show that they did not consider cholera evidently contagious. These statements are all authenticated by Mr. Gibsone, the British Consul in this place, who vouches for the high professional character of these gentlemen.

1st. Extract from a Report drawn up for Dr. Hamett, by Dr. Baum, physician-general of the Town Hospital in Danzig:

'As to the contagion of cholera morbus, there is a great contest among our physicians, five being for, and twenty against contagion.

'In my experience, the disease certainly proved not to be contagious. There were five waiters always near the patients: eight men were employed in rubbing and bathing; nine medical men visited the patients, of whom one was always in the room in the day-time; two watching every night: no one of these twenty-two persons fell ill. Many patients suspected to labour under this disease were brought into the same wards, but the disease did not appear in them.

'Although the medical assistants, and the eight labourers were constantly going from the cholera wards to the other patients, there were but five patients who caught the disease in my hospital. Now the number of patients on June 1st, amounted to three hundred; three hundred were received in the month of June, so that out of six hundred patients only five got the disease, which is much less than the common number of patients taken ill with cholera, among an equal number of poor, wretched, unhealthy people in the town. Of these five persons only two became ill in the same room; the other three being scattered over the whole large establishment.

'The disease made its appearance in Danzig without communication with any unhealthy place. The only ship about that time coming from such a place was the Monna, Captain Brandt, from Riga, who arrived the 30th of May, two days* after the first appearance of the disease.

* It has been proved to be three days after the first appearance of the disease.

'It did not spare institutions which were perfectly shut against any communication with the town. In the public gaol, one person; and in the two institutions for poor children and orphans, some children were attacked. It in the same way broke out in Elbing, ten German miles from Danzig, after the quarantine had been carefully kept up between the two places.

'It was preceded by a remarkable change of weather; the temperature often differing in some hours' time nearly ten degrees R. It was preceded by immense quantities of fish being caught, and of so low a price, that all the poor people had lived the whole months of April and May on almost nothing else. *Esox belone* and *clupea sprattus* were the most common.

W. Baum, M.D., Physician of the Town Hospital.'

*Danzig, Aug.* 1*st,* 1831.

This is the writing and signature of Doctor Baum.

Alexander Gibsone, British Consul.

2. Extract from a Communication to Dr. Hamett by Dr. Baum, dated Danzig, September 17th, 1831, which is authenticated by Mr. Gibsone:

'The last three striking cases of the epidemic cholera that occurred in the Town Hospital at Danzig, remained in their beds in the common wards, and there appeared no spreading of the disease; no person was attacked in the hospital after them.'

3. In the Military Cholera Hospital in Danzig, have been received from the 8th of July to the 28th of August, 1831, inclusive, ninety-eight cholera patients:

For the superintendance of the Cholera Hospital—A. One regimental physician. B. One assistant-surgeon of the first class. C. Four sick attendants. D. One porter.

For the superintendance of the Contumace division of the Institution—A. One inspector of wards. B. One assistant-surgeon. C. One cook-maid. D. Four to five attendants, according to the number of those in the Contumace. E. Two sentinels, who are regularly relieved from the military guard in the Hospital.

A sick attendant in the Cholera Hospital was attacked with the *cholera cardialgica*, but was cured in three days.

The which testifies,

Dr. Sinogowitz, Regimental Physician,
*Danzig, Aug* 29*th,* 1831. Physician of the Cholera Hospital.

This is a correct translation of the certificate drawn out, and signed by Dr. Sinogowitz, Regimental Physician, &c.

*Danzig, Aug.* 30*th,* 1831. Alex. Gibsone, British Consul.

4. Between the 1st of July and 30th of August, 1831, there have been received into Cholera Hospital No. 2, one hundred and forty patients affected with cholera.

In attendance on the patients there have been—A. One physician. C. One barber-surgeon, and superintendent. E. One assistant-surgeon. A. One matron. C. Six men in immediate attendance on the sick. D. Three servants of all work. E. Two porters.

Besides these, there have been two foreign physicians in daily attendance on the sick for one month.

Of all these there have been only three of the sick attendants slightly attacked with the primary symptoms of cholera, owing entirely to fatigue and profuse perspiration, and catching cold in consequence.

I certify the same, this 30th day of August, 1831, in Danzig.

Edward Otto Dann, H. M. D.
and Physician of Cholera Hospital No. 2, in Danzig.

The foregoing is the hand-writing and signature of Doctor Dann, II., given to Doctor Hamett.

*Danzig, Sept.* 1*st,* 1831. Alex. Gibsone, British Consul.

It will be proper to state here, that in Cholera Hospitals No. 2 and No. 3, the sick attendants commonly slept in their clothes, and were continually subject to profuse perspiration, in consequence of being exposed to the heat and vapour of the hot-baths, that were incessantly administered. Hence, from their severe duties and close confinement, they always looked pale, and were occasionally indisposed. The washing in these Cholera Hospitals was great and incessant. In No. 3 it was done under a shed.

I have particularly inquired of medical men who have attended patients affected with cholera, and patients affected with other diseases at the same time, and have not heard of one instance of cholera taking place among the patients or their families in consequence. Dr. Dann, senior, who has had more general practice in cholera, other diseases, and *midwifery,* than any other physician in Danzig, has assured me that there has been no instance of the disease being communicated by himself or his clothes; and that he has not heard of any by other general practitioners like himself.

I could have procured additional evidence in this respect from all the medical men at Danzig, who have had experience in cholera.

There have been instances of a wife, or child, or both having slept in the same bed with a cholera patient, without any bad consequences ensuing; and the instances of escape of whole families shut up in small rooms with cholera patients have been numerous. The four first cases of the epidemic in Danzig, the Separate List, and the able communications to me from that observant, talented, and conscientious Gentleman, Mr. Gibsone, the British Consul at Danzig, will afford proofs of these statements.

In the history of the first appearance, and subsequent spread of the disease, no evidence of contagion has appeared; while in the police reports in the General and Separate Lists, a probable, or predisposing cause has been assigned for almost every case of it, during the first eight weeks of the epidemic. The facts here adduced, with all those relating to the shipping, harbour, &c. &c., which are embodied in the history of the disease, prove indeed extraordinary escapes from it.

Finally, it has been evinced, that the disease is not necessarily produced, either by inoculation of matter from a recent subject of it, or from inhalation of the *effluvia* arising from one left exposed for several days in a putrid state, swarming with insects, in a neglected cholera burying-ground contiguous to a populous village; the former, particularly in my own person, twice from casualty at dissection; the latter from the fact itself contained in an accurate statement, authenticated as usual by Mr. Gibsone.

If the disease, however, be contagious, in the personal communicable sense of the term, independently of what I conceive to be the adequate cause of it,—namely, a modification of infectious *miasmata,* specifically operating on certain constitutions and habits, rendered more or less susceptible in the manner I have specified in the history of the disease; and the numberless instances of escape, which I have made to appear, are not sufficient to overpower the argument of contagion, deduced simply from the gradual, but irregular and uncertain appearance, and spread of the disease throughout the world, they at least deprive it of a great part of its terror.

JOHN HAMETT, M.D.

☞ Our readers will see that the foregoing most valuable document contains the only minute and circumstantial account, semeiological and pathological, of the Continental cholera, which has yet appeared. If Dr. Hamett has evinced *liberal* ideas in respect to the propagation of the disease, we hope a *liberal* Government will not pass over unrewarded, the only *Naval* Medical Officer which it has employed in this investigation. *Nous verrons !*—ED.

